# Li^+^ Diffusion in Li_n_CoNb_2_O_6_ (0 < n ≤ 6) Anode with High Capacity Density: Fast Kinetics and Mechanistic Insights

**DOI:** 10.1002/advs.202416001

**Published:** 2025-03-19

**Authors:** Yimo Xiang, Shaowen Tan, Jingxian Yu, Shengping Wang

**Affiliations:** ^1^ Faculty of Materials Science and Chemistry China University of Geosciences Wuhan 430074 China; ^2^ Guangxi Key Laboratory of Electrochemical and Magneto‐Chemical Functional Materials College of Chemistry and Bioengineering Guilin University of Technology Guilin 541004 China; ^3^ School of Chemistry Physics and Earth Sciences The University of Adelaide Adelaide SA 5005 Australia

**Keywords:** anode, direct‐hoping, ion diffusion, knock‐off

## Abstract

The potential of high power/capacity density and Li^+^ solid diffusion mechanisms of niobium‐based binary metal oxide (CoNb_2_O_6_) anode material are investigated by combining high‐rate Nb_2_O_5_ with the redox‐active 3d transition metal Co. CoNb_2_O_6_ exhibited exceptional rate capability and cycling stability, which is attributed to anisotropic expansion during cycling and dual diffusion mechanisms at high and low lithium concentrations. The anisotropic expansion of crystals ensures structural stability, whereas the organic combination of a direct‐hopping diffusion mechanism in Li_n_CoNb_2_O_6_ (0 ≤ n ≤ 3) and a knock‐off diffusion mechanism in Li_n_CoNb_2_O_6_ (3 < n ≤ 6) based on the nudged elastic band (NEB) calculations reveals rapid Li^+^ solid diffusion and excellent rate performance during lithiation/delithiation. The electrochemical performance of CoNb_2_O_6_ also depends on its morphology, where different structures modulate synergistic Nb and Co interactions, influencing Li^+^ diffusion in the Nb layers. Specifically, the micron‐scale structure formed by secondary particle attachment (CoNb_2_O_6_‐MP) provides space for anisotropic expansion, fully utilizing the dual ion diffusion mechanism, enhancing diffusion efficiency, and delivering both high‐capacity density and excellent rate performance. This work not only introduces CoNb_2_O_6_ with superior electrochemical properties but also provides insights into the solid diffusion mechanisms under various lithium concentrations, offering a foundation for designing electrode materials with enhanced ion diffusion pathways.

## Graphical abstract

CoNb_2_O_6_ with 6 Li^+^/e⁻ reaction and high theoretical capacity density of 472 mA g^−1^, exhibited exceptional rate capability and cycling stability, which is attributed to dual Li^+^ solid diffusion mechanisms, namely direct‐hopping mechanism in Li_n_CoNb_2_O_6_ (0 ≤ n ≤ 3) and knock‐off mechanism in Li_n_CoNb_2_O_6_ (3 < n ≤ 6).

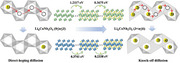

## 1 Introduction

With the rapid increase in the global population and the intensification of the greenhouse effect, the demand for clean and renewable energy continues to grow.^[^ ^1^, ^2^ ^]^ Lithium‐ion batteries (LIBs), which have numerous technical advantages, remain a primary research focus, particularly for improving energy density, power density, and safety. Graphite, a commercial anode material, offers a high theoretical capacity (372 mAh g^−1^), structural stability, and low cost.^[^ ^3^, ^4^ ^]^ Its low lithiation potential (≈0.1 V vs Li^+^/Li) promotes lithium dendrite formation, leading to safety concerns. Moreover, the limited kinetics at high power levels make it insufficient to meet growing energy demands.^[^ ^5^, ^6^, ^7^ ^]^ As an alternative, spinel‐type Li_4_Ti_5_O_12,_ with a theoretical capacity of 175 mAh g^−1^, has been recognized for its “zero‐strain” properties, excellent cycling stability,^[^ ^8^, ^9^ ^]^ and high lithiation potential (≈1.55 V vs Li^+^/Li). The high lithiation potential prevents solid electrolyte interphase (SEI) formation and inhibits lithium dendrites, improving safety, although at the expense of reduced energy density.^[^ ^10^ ^]^ Therefore, identifying new anode materials with superior rate capability, extended cycle life, and an optimal voltage range has become critical for advancing fast‐charging batteries.^[^ ^11^ ^]^

Niobium‐based oxides offer excellent electrochemical performance in terms of rate capability, cycling stability, and safety. Nb_2_O_5_, a niobium‐based unary metal oxide, has a strong rate performance, and its low theoretical capacity (≈200 mAh g^−1^) is unsatisfactory.^[^ ^12^, ^13^ ^]^ Combining Nb_2_O_5_ with redox‐active 3d/5d transition metals to form binary metal oxides (M‐Nb‐O) yields higher capacities and improved rate performance.^[^ ^14^ ^]^ M‐Nb‐O materials with Wadsley‐Roth shear structures are composed of m×n×∞ reo3‐type blocks, such as TiNb_2_O_7_,^[^ ^15^, ^16^ ^]^ Ti_2_Nb_10_O_29_,^[^ ^17^, ^18^ ^]^ W_5_Nb_16_O_55_,^[^ ^19^ ^]^ WNb_12_O_33_,^[^ ^20^ ^]^ and FeNb_11_O_29_.^[^ ^21^ ^]^ It has garnered significant attention for its high‐voltage operation, which prevents SEI formation and dendrite growth. However, this approach comes at the cost of reduced energy density, low active site utilization, high production costs, and complex synthesis. In this study, hydrothermally synthesized CoNb_2_O_6_ exhibited a high theoretical capacity (472 mAh g⁻^1^ with 6 Li^+^) and excellent rate performance (the 1,000th discharge capacities of ≈260 mAh g^−1^ at 2.14 C (1 mA cm^−2^, 1 C = 0.467 mA cm^−2^ = 472 mA g^−1^) and ≈200 mAh g^−1^ at 10.70 C (5 mA cm^−2^) and is a highly promising material for LIBs. Its robust 3D structure ensures stability during cycling, whereas the ≈0.7 V lithiation potential minimizes the risk of dendrite formation.

This study reveals a Li^+^ bimodal diffusion mechanism in CoNb_2_O_6_, which is distinct from shear‐based diffusion in Wadsley‐Roth structures and offers a new perspective on the high‐rate performance of niobium‐based binary oxides. Density functional theory (DFT) calculations revealed that at low lithium concentrations (Li_n_CoNb_2_O_6_, 0 ≤ n ≤ 3), Li⁺ diffuses via direct‐hopping in the Co layer with a low migration barrier of 0.3741 eV. Different from the previously reported direct‐hopping diffusion mechanism in the full lithiation process,^[^ ^22^, ^23^ ^]^ although CoNb_2_O_6_ with low lithium insertion concentration (Li_n_CoNb_2_O_6_, 0 ≤ n ≤ 3) still relies on the direct‐hopping mechanism to transport Li^+^, when CoNb_2_O_6_ has a high lithium insertion concentration (Li_n_CoNb_2_O_6_, 3 < n ≤ 6), the Li^+^ solid transport process is a knock‐off mechanism. Continuous channels were formed in both the Nb and Co layers in Li_n_CoNb_2_O_6_ (3 < n ≤ 6), reducing the migration barriers to 0.3675 and 0.2338 eV, respectively, enhancing the diffusion efficiency.

Additionally, morphology plays a critical role in the diffusion process. By controlling the morphology of CoNb_2_O_6_ through hydrothermal synthesis, micro rods (CoNb_2_O_6_─MR), micro cross‐structures (CoNb_2_O_6_‐MC), and microspheres (CoNb_2_O_6_‐MP) of CoNb_2_O_6_ were obtained. The results show that CoNb_2_O_6_─MR and CoNb_2_O_6_‐MC hinder Li⁺ intercalation in the Nb layers, degrading the electrochemical performance. However, CoNb_2_O_6_‐MP fully utilizes the bimodal mechanism, achieving high capacity and excellent rate performance. This study highlights CoNb_2_O_6_ bimodal diffusion as the key to its high‐rate performance, explores the role of morphology in Li⁺ intercalation, and offers new insights for developing fast‐charging anode materials.

## 2 Results and Discussion

### 2.1 Solid Diffusion Mechanism of CoNb_2_O_6_

#### 2.1.1 High‐Rate Characteristics of CoNb_2_O_6_

**Figure 1:**
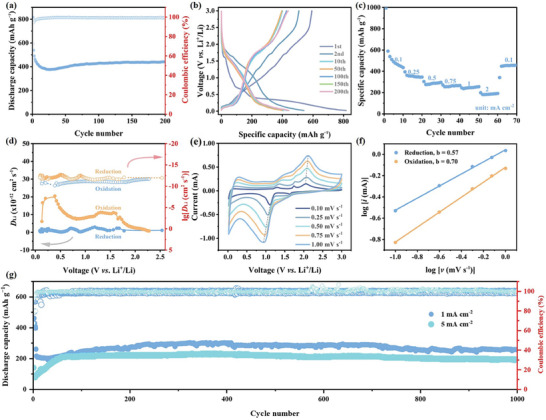
Electrochemistry performance of CoNb_2_O_6_‐MP. a) Cycling performance at 0.1 mA cm^−2^, b) charge/discharge curves at 0.1 mA cm^−2^, c) rate performance, d) Li^+^ diffusion coefficients at different voltages, e) CV curves, f) b‐values at the redox peaks, and g) cycling performance at 1 (2.14 C), and 5 mA cm^−2^ (10.70 C).

CoNb_2_O_6_‐MP formed through the aggregation of secondary nanoparticles (Figure S1–S3, Table S1, Supporting Information), facilitating the exposure of more active sites and enhancing interaction with the electrolyte. This significantly increased the energy density and reactivity, resulting in excellent electrochemical performance for LIB anode materials (**Figure** **1**). Compared with Nb_2_O_5_, the incorporation of cobalt enhanced the capacity characteristics of CoNb_2_O_6_, thereby improving the energy storage capacity (Figure S4, Supporting Information). CoNb_2_O_6_‐MP exhibited excellent cycling stability, with a capacity of 442.3 mAh g^−1^ after 200 cycles at 0.1 mA cm^−2^. Additionally, CoNb_2_O_6_‐MP retained the inherent rate performance advantage of Nb_2_O_5_, with stable and recoverable electrochemical behavior after rate testing (Figure 1c; Figure S5a,b, Supporting Information). The 1,000th discharge capacities of CoNb_2_O_6_‐MP were ≈260 mAh g^−1^ at 2.14 C and 200 mAh g^−1^ at 10.70 C (Figure 1g). After 1000 cycles, the crystal structure of CoNb_2_O_6_‐MP remained intact (Figure S6, Supporting Information). These results indicate that CoNb_2_O_6_‐MP has significant potential for fast charge/discharge applications, making it a highly promising high‐performance energy storage material.

Cyclic voltammetry (CV) tests at varying scan rates were used to explore CoNb_2_O_6_‐MP energy storage kinetics, with the b‐value serving as a key indicator of the underlying transport kinetics (Figure 1f; Figure S7, Supporting Information).^[^ ^24^, ^25^ ^]^ A b‐value of 0.5 suggested solid diffusion, whereas values closer to 1 indicated surface diffusion. The b‐values of CoNb_2_O_6_‐MP were 0.57 for the reduction peaks and 0.7 for the oxidation peaks, indicating that the electrode reaction kinetics were governed primarily by solid diffusion. The galvanostatic intermittent titration technique (GITT) results (Figure 1d; Figure S5c, Supporting Information) confirmed a high Li⁺ diffusion coefficient (10^−11^−10^−12^ cm^2^ s^−1^), highlighting strong ion transport.^[^ ^23^, ^26^ ^]^ Electrochemical impedance spectroscopy (EIS) measurements revealed a significant decrease in the charge transfer resistance (*R*_ct_) at the open‐circuit voltage (OCV) from 846.8 ohm before the first cycle to 39.97 ohm after 200 cycles, indicating that Li⁺ charge transfer was enhanced (Figure S8 and Table S2, Supporting Information). CoNb_2_O_6_‐MP retained 182 mAh g^−1^ after 150 cycles in a full cell with LiFePO_4_ (Figure S9, Supporting Information), demonstrating strong reversibility and stability and confirming its potential for LIB applications.

#### 2.1.2 Lithiation Behavior in CoNb_2_O_6_ Crystals

**Figure 2:**
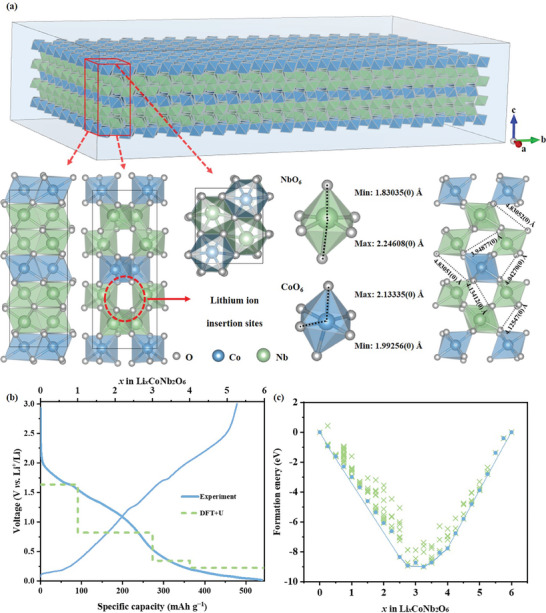
a) Schematic diagram of the crystal structure, b) lithiation plateau, and c) hull diagram for CoNb_2_O_6_.

CoNb_2_O_6_‐MP, a binary niobium‐based oxide, features an orthorhombic structure (Pbcn) with NbO_6_ and CoO_6_ octahedra forming layered sandwich configurations (**Figure** **2**a). These layers create zigzag ion chains and voids for Li^+^ storage, enabling efficient migration along the a‐axis, which is critical for high‐rate performance. The stable 3D network of the NbO_6_ and CoO_6_ octahedra, linked by shared oxygen atoms, enhanced the structural stability and cycling durability, preventing degradation during Li^+^ insertion/extraction. The high reactivity of Co and Nb in CoNb_2_O_6_‐MP resulted in complex reactions. In the CV curves, the redox peak at 1.63/1.75 V corresponded to Nb^5+^/Nb^4+^, and that at 1.08/2.05 V corresponded to Co^2+^/Co^1+^ (Figure S10, Supporting Information). The oxidation peak at 1.30 V and low‐voltage phenomenon (0.45–0.02 V) involve Nb^4+^/Nb^3+^ and partial Nb^3+^/Nb^2+^ transitions.^[^ ^27^, ^28^ ^]^ The reduction peak at 0.78 V in the first cycle was attributed to Co/Li_2_O and SEI formation.^[^ ^29^ ^]^ Low‐voltage reactions were prominent in the first cycle, which is consistent with the DFT+U results (Figure 2b) and d*Q*/d*V* curves (Figures S11 and S12, Supporting Information). XPS revealed that the Nb^5+^ and Co^2+^ peaks shifted during charging, with the change in Nb^5+^ linked to oxygen vacancies and Co^2+^ partially converting to metallic Co, leading to irreversible capacity loss, which is consistent with the CV data (Figure S13, Supporting Information).

Simulations of the CoNb_2_O_6_ structure revealed that it could accommodate 24 Li^+^ ions per Co_4_Nb_8_O_24_ unit cell (Figure 2c; Figure S14 and Table S3, Supporting Information). This value was significantly greater than those of graphite and Li_4_Ti_5_O_12_, highlighting its potential for practical applications. Lithiation in CoNb_2_O_6_ followed a specific sequence; initially, Li^+^ occupied the O8 octahedral sites in Co layer‐4, facilitated by the weaker Co‐O bond (1.99256 Å, longer than Nb‐O 1.83035 Å) and larger entry aperture, reducing spatial hindrance. Subsequently, Li^+^ filled the O3 octahedral sites in Nb layer‐2 to minimize repulsion. As lithiation continued, Li^+^ occupied all available octahedral sites, forming Li_3_CoNb_2_O_6_. Once these sites were filled, Li^+^ transitioned to the tetrahedral sites in Co layer‐4 and Co layer‐1. After the Co layers were filled at the 4 tetrahedral sites, Li^+^ moved to the 8 tetrahedral sites in the Nb layer, ultimately forming Li_6_CoNb_2_O_6_. Despite some structural distortion during lithiation, the overall 3D framework remained stable, demonstrating the excellent reversibility of lithiation/delithiation for CoNb_2_O_6_.

The reversibility and structural stability of CoNb_2_O_6_‐MP were confirmed via ex situ X‐ray diffraction (XRD) (Figure S15 and Tables S4 and S5, Supporting Information). During lithiation, the diffraction peaks for the (310) and (311) crystal planes shifted to lower angles, indicating increased interlayer spacing due to the reduction of Nb^5+^ to Nb^2+^. Upon Li^+^ extraction, these peaks shifted back, indicating high reversibility. The consistent peak shifts without new peak formation suggested a single‐phase solid‐solution reaction mechanism, which is essential for rapid lithiation/delithiation. Notably, CoNb_2_O_6_‐MP exhibited anisotropic expansion, ranging from −0.23821% to 0.050645%, which was attributed to the synergy between Nb and Co. This behavior enhances structural stability and Li^+^ diffusion, with negative expansion along the a‐axis shortening the Li⁺ diffusion path, underscoring its potential as a high‐performance anode material.

#### 2.1.3 Diffusion Behavior of CoNb_2_O_6_

##### Direct‐Hopping Diffusion Mechanism of Li_n_CoNb_2_O_6_ (0 ≤ n ≤ 3)

**Figure 3:**
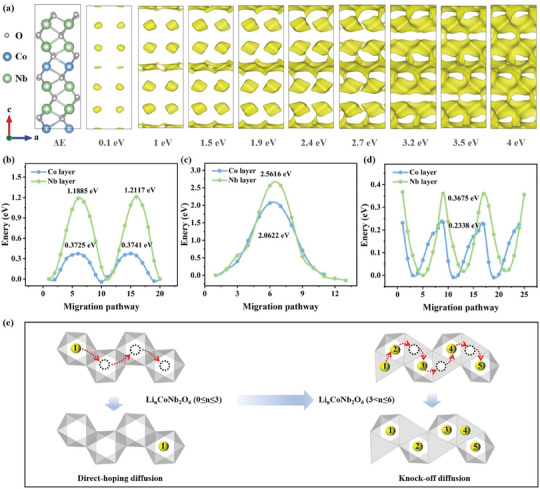
Calculation of the lithiation process for CoNb_2_O_6_. a) BVES energy landscape for Li^+^ diffusion, the migration energy barriers for Li^+^ diffusion along the Co and Nb layers b) via the direct‐hopping mechanism at low lithium concentrations, c) via the direct‐hopping mechanism at high lithium concentrations, d) via the knock‐off mechanism at high lithium concentrations, e) schematic of the dual diffusion mechanism in the CoNb_2_O_6_ crystal.

The bond valence energy site (BVES) method was used to explore Li^+^ diffusion in CoNb_2_O_6_.^[^ ^30^, ^31^ ^]^ BVES revealed that Li^+^ is primarily distributed in octahedral voids, with the Co layer forming a continuous Li^+^ migration pathway (**Figure** **3**a). This suggested that the Co layer offered low‐energy barriers for Li^+^ migration, enabling rapid Li^+^ transport. As lithiation increased, continuous Li^+^ migration pathways also emerged in the Nb layer, eventually forming a 3D network within the CoNb_2_O_6_ crystal. While BVES provided an initial understanding of ion migration in CoNb_2_O_6_, further detailed and accurate studies using DFT are needed.

The diffusion behavior of Li^+^ along the a‐ and c‐axes in the Nb and Co layers of CoNb_2_O_6_ was calculated via the nudged elastic band (NEB) method.^[^ ^32^ ^]^ Li^+^ was placed in octahedral voids of the Nb and Co layers in the Co_4_Nb_8_O_24_ crystal, with migration along the a‐axis set as the final state (Figure S16, Supporting Information). Li^+^ migration in both the Nb and Co layers followed a direct‐hopping mechanism. The migration energy barrier was 1.2117 eV through the Nb layer but was significantly lower at 0.3741 eV in the Co layer. At low lithium concentrations, the high barrier in the Nb layer confined Li^+^ diffusion, resulting in fixed storage sites and a plateau voltage in the charge/discharge curve (Figure 3b).^[^ ^23^ ^]^ In contrast, the lower barrier enabled continuous diffusion pathways at the Co layer, favoring rapid lithiation/delithiation, which is consistent with the BVES results. To further investigate the potential for 3D diffusion in CoNb_2_O_6_, simulations along the c‐axis revealed higher, irregular migration barriers, indicating thermodynamically unfavorable Li^+^ migration in the c‐axis direction (Figure S17, Supporting Information). DFT calculations provided deeper insight into the Li^+^ diffusion mechanism in CoNb_2_O_6_. In the CoNb_2_O_6_ crystal, when the lithiation amount was 0 ≤ n ≤ 3, Li^+^ occupied octahedral voids and diffused through a single pathway via direct hopping. The Co layer has a low migration barrier of 0.3741 eV, which is lower than those of TiO_2_, TT‐Nb_2_O_5_, T‐Nb_2_O_5_, and NiNb_2_O_6_ (Table S6, Supporting Information). This suggests that CoNb_2_O_6_ has strong potential for Li^+^ diffusion, making it promising for good electrochemical performance.

##### Knock‐Off Diffusion Mechanism of Li_n_CoNb_2_O_6_ (3 ≤ n ≤ 6)

DFT+U calculations, which closely match the experimental data, revealed that the lithiation process of CoNb_2_O_6_ exceeded 3 Li^+^. This suggested that Li^+^ diffusion in CoNb_2_O_6_ involved migration between both tetrahedral and octahedral voids, not just the latter. To delve deeper into lithiation kinetics, the study extended beyond conventional low‐concentration simulations as M‐Nb‐O (Ni, Co).^[^ ^22^, ^23^ ^]^ At high lithium concentrations, Li^+^ is present in unoccupied tetrahedral voids at the Co and Nb layers of Li_12_Co_4_Nb_8_O_24_, with all octahedral voids fully occupied (Figure S18a, Supporting Information). The final state involves Li^+^ migrating along the a‐axis to the next tetrahedral site (Figure S18b, Supporting Information). The NEB calculations revealed that the high energy barriers for direct‐hopping between tetrahedral voids in the Nb and Co layers were 2.5616 and 2.0622 eV, respectively (Figure 3c). These findings contradict BVES predictions of continuous diffusion pathways and are unfavorable for CoNb_2_O_6_ in achieving optimal rate performance.

Based on these findings, it was hypothesized that at high lithium concentrations, Li^+^ migration in CoNb_2_O_6_ followed a nontraditional hopping mechanism. Considering the increased atomic repulsion at high lithium concentrations, the knock‐off mechanism, which has been observed in inorganic solid electrolytes such as Li_10_SiP_2_S_12_, Li_5_La_3_Ta_2_O_12_, and Na_3_Zr_2_Si_2_PO_12_, was described previously.^[^ ^33^, ^34^, ^35^, ^36^ ^]^ The mechanism has been confirmed in electrode materials, where Li^+^ migrates through a cooperative knock‐off process.^[^ ^37^ ^]^ In this process, a newly intercalating Li^+^ occupies a tetrahedral site, displacing a nearby Li^+^ from an octahedral site to a vacant tetrahedral site. To validate this mechanism at high lithium concentrations, NEB calculations were performed for the initial, transition, and final states in the Nb and Co layers (Figure S18c,d, Supporting Information). The results show significantly reduced migration barriers of 0.3675 eV in the Nb layer and 0.2338 eV in the Co layer, which are much lower than those for the direct‐hopping mechanism (Figure 3d). This suggests that once octahedral voids are fully occupied, Li^+^ diffusion shifts from the direct‐hopping mechanism to the knock‐off mechanism, enhancing the Li^+^ lithiation/delithiation kinetics (Figure 3e; Figures S7 and S19, Supporting Information). The transition is influenced by the Li^+^ distribution and interactions among ions. In the knock‐off diffusion chain, two adjacent Li^+^, Li1 and Li2, occupy a high‐energy tetrahedral site (excited‐state Li^+^) and a low‐energy octahedral site (ground‐state Li^+^), respectively. Li1 spontaneously slides into the octahedral site, replacing the ground‐state Li^+^, while Li2 transitions to the tetrahedral site, becoming the new excited‐state Li^+^. The Li^+^ diffusion is controlled by the energy transfer from the low‐energy octahedral position to the high‐energy tetrahedral position. Compared to the independent direct‐hopping mechanism, the knock‐off mechanism leverages coulomb interactions between ground‐state and excited‐state Li^+^, lowering the energy barrier for the transition. This facilitates faster solid‐state diffusion, whereas the direct‐hopping mechanism, lacking the interaction, exhibits a significantly higher migration barrier. XRD analysis revealed that the CoNb_2_O_6_ crystal underwent significant anisotropic expansion during Li^+^ diffusion, which was closely related to the diffusion process. This structural feature helped dynamically adjust the diffusion channels during Li^+^ intercalation, and the optimization supported rapid lithium transport, effectively leveraging the benefits of 1D diffusion. This dual diffusion mechanism highlights the adaptability of the CoNb_2_O_6_ structure and explains the formation of continuous ion diffusion pathways between the Nb and Co layers.

Physicochemical characterization, DFT calculations, and BVSE analysis conclusively demonstrated that CoNb_2_O_6_ is a highly promising material for LIBs. Its high capacity results from its excellent lithiation capability, which is stable because of its robust 3D framework. Notably, the rate performance stemmed from a dual diffusion mechanism within CoNb_2_O_6_. For Li_n_CoNb_2_O_6_ (0 ≤ n ≤ 3) with low lithium concentrations, Li^+^ rapidly migrated through the Co layer via a direct‐hopping mechanism. As the lithium concentration increased (Li_n_CoNb_2_O_6_, 3 < n ≤ 6), the migration shifted to a cooperative knock‐off mechanism, enabling CoNb_2_O_6_ to maintain efficient Li^+^ diffusion. This finding deepened our understanding of Li^+^ migration in niobium‐based oxides and offered guidance for designing more durable, high‐performance LIBs.

### 2.2 Morphology and Solid Diffusion Behavior of CoNb_2_O_6_

#### 2.2.1 Influence of CoNb_2_O_6_ Morphology on Rate Performance

Analysis of the different morphologies of CoNb_2_O_6_─MR, CoNb_2_O_6_‐MC, and CoNb_2_O_6_‐MP revealed significant variations in their electrochemical performance (Figures S1 and S3, Supporting Information). The initial discharge/charge capacities of CoNb_2_O_6_─MR, CoNb_2_O_6_‐MC, and CoNb_2_O_6_‐MP were 469.9/296.9, 737.3/516.3, and 817.3/592.4 mAh g^−1^, respectively (Figure S11, Supporting Information). The 100th discharge capacities were 186, 277, and 431 mAh g^−1^, respectively, highlighting the impact of morphology on performance. CoNb_2_O_6_‐MP exhibited the best electrochemical performance, attributed to its unique microstructure of nanoscale secondary particles, which enhanced ion and electron transport. These findings suggest that Li^+^ diffusion in CoNb_2_O_6_ depends on both the crystal structure and morphology. Further analysis of the CV curves revealed that CoNb_2_O_6_‐MC and CoNb_2_O_6_‐MP had similar charge/discharge profiles, while CoNb_2_O_6_─MR presented weaker, diminishing redox peaks for Nb^5+^/Nb^4+^ and Nb^4+^/Nb^2+^ over cycles, indicating underutilization of Nb and resulting in a lower capacity. Further analysis of the CV curves for different CoNb_2_O_6_ morphologies revealed both similarities and notable distinctions (Figure S20, Supporting Information).

These findings highlight the crucial role of morphology in Li^+^ diffusion and electrochemical performance. Morphology impacts surface characteristics, charge transfer, and Li^+^ transport pathways, which together determine the lithium storage capacity and cycling stability. A thorough investigation into how morphology affects Li^+^ diffusion is crucial for optimizing electrode materials. This understanding will guide the design of electrodes with higher energy densities, faster charge/discharge rates, and better cycling stability, supporting the advancement and wider application of LIB technology.

#### 2.2.2 Influence of CoNb_2_O_6_ Morphology on Dual Diffusion Behavior

The complex Li^+^ diffusion kinetics in CoNb_2_O_6_ were systematically analyzed through BVES, DFT+U, and NEB calculations. The electrochemical differences across various CoNb_2_O_6_ morphologies revealed distinct Li^+^ diffusion behaviors. Further CV analysis revealed that the Nb^5+^/Nb^4+^ and Co^2+^/Co^1+^ redox reactions corresponded to Li^+^ intercalation into the octahedral voids of the Nb and Co layers, respectively. During the 0 ≤ n ≤ 3 stage, the higher migration energy barrier in the Nb layer indicated that the rate performance was driven primarily by diffusion in the Co layer. As the Li^+^ concentration increased, the Nb^4+^/Nb^2+^ redox reaction shifted to lithium intercalation into tetrahedral voids, transitioning the migration mechanism from direct‐hopping to a more efficient knock‐off mechanism and reducing the migration barrier. Li^+^ behavior was evident in the d*Q*/d*V* profiles, particularly at ≈0.2 V (Figure S12, Supporting Information). In CoNb_2_O_6_─MR, the CV and d*Q*/d*V* profiles initially showed distinct Li^+^ intercalation features, but redox peaks related to b^5+^/Nb^4+^ weakened with cycling, leaving only those for Co^2+^/Co^1+^. The low‐voltage peaks for tetrahedral void intercalation persisted, with Li^+^ extraction and Co^1+^/Co^2+^ oxidation merging into broad peaks. In CoNb_2_O_6_‐MP, the peaks for lithium intercalation into both octahedral and tetrahedral sites merged during reduction and oxidation, which is consistent with pseudocapacitive behavior,^[^ ^38^ ^]^ underscoring the potential of CoNb_2_O_6_ as a high‐performance energy storage material.

CoNb_2_O_6_─MR has a unidirectional microrod structure with a smooth and dense surface, restricting Li^+^ diffusion to a certain extent and hindering rapid insertion/extraction. According to the d*Q*/d*V* curves, only Li^+^ migration in the Co layer octahedral voids and cooperative tetrahedral migration remained effective during the later cycling stages, resulting in a low capacity of 186 mAh g^−1^. CoNb_2_O_6_‐MC, featuring a multidirectional micro cross‐structure, also had a smooth, dense surface that limited Li^+^ mobility to some certain degree, causing the octahedral migration peaks of the Nb layer to disappear over time. However, some Li^+^ remained in the Nb layer octahedral voids, acting as structural pillars. Although CoNb_2_O_6_‐MC performed better than CoNb_2_O_6_─MR, with a capacity of 277 mAh g^−1^, it still fell short of ideal performance. In contrast, CoNb_2_O_6_‐MP, composed of secondary nanoparticles, offered space for lattice expansion, which facilitated dual diffusion behavior. However, the stable crystal structure requires an initial activation process to release its full capacity potential. Similar behaviors were observed for CoNb_2_O_6_,^[^ ^39^, ^40^ ^]^ MnNb_2_O_6_,^[^ ^41^ ^]^ NiNb_2_O_6_,^[^ ^42^, ^43^ ^]^ ZnNb_2_O_6_,^[^ ^44^ ^]^ and CuNb_2_O_6_, where the capacity initially declined before recovering. After rate cycling, when CoNb_2_O_6_ returned to 0.1 mA cm^−2^, the capacity stabilized at 468 mAh g^−1^ after 160 cycles, achieving effective activation and fully realizing the CoNb_2_O_6_ capacity (Figure S5a, Supporting Information).

**Figure 4:**
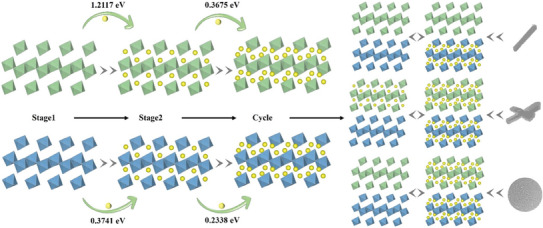
Li^+^ diffusion mechanisms in CoNb_2_O_6_ with different morphologies.

The high discharge capacity and cycling stability of CoNb_2_O_6_‐MP highlight the positive impact of morphology on Li^+^ transport and structural stability. Li^+^ diffusion in CoNb_2_O_6_ is influenced by both the crystal structure and morphology. The different morphologies of CoNb_2_O_6_ affect lithiation reactions, leading to varied diffusion behaviors in the Nb and Co layers. The microrod and micro cross‐structures restrict Li^+^ intercalation in the Nb layer, reducing the diffusion and electrochemical performance (**Figure** **4**). In contrast, the microsphere structure effectively leveraged the CoNb_2_O_6_ dual diffusion mechanism, resulting in a high capacity and excellent rate performance. This study reveals how different morphologies impact CoNb_2_O_6_ performance and highlights the unique advantages of its crystal structure in LIB anode applications. This study offers a new perspective on how the high‐rate performance of niobium‐based binary materials is derived from their dual diffusion mechanisms.

## 3 Conclusion

CoNb_2_O_6_ is a niobium‐based binary metal oxide anode material with anisotropic expansion properties, offering high capacity and excellent rate performance. Its 6‐electron reaction leads to a higher capacity, whereas the intercalation structure and solid‐solution reaction enable fast Li^+^ diffusion. The dual diffusion mechanism contributes to its rate performance; at low lithium concentrations, Li^+^ primarily diffuses via direct‐hopping, with a migration barrier as low as 0.3471 eV in the Co layer. At higher lithium concentrations, the mechanism shifts to a knock‐off transition, further lowering the migration barrier to 0.2338 eV in the Co layer and 0.3675 eV in the Nb layer. The synergistic interaction between the Nb and Co layers causes anisotropic expansion during the charge/discharge process, optimizing diffusion pathways for fast Li^+^ transport and emphasizing the benefits of 1D diffusion. In addition to the crystal structure, the morphology of CoNb_2_O_6_ influences Li^+^ diffusion, particularly the interaction between Nb and Co. CoNb_2_O_6_‐MP maintains a discharge capacity of 442.3 mAh g^−1^ (at 0.214 C) after 200 cycles, 260 mAh g^−1^ (at 2.14 C) and 200 mAh g^−1^ (at 10.70 C) after 1000 cycles. With physicochemical characterization and DFT and BVSE calculations, CoNb_2_O_6_ was confirmed to be a promising material that is essential for meeting the market demand for high energy density and power density in LIBs.

## Conflict of Interest

The authors declare no conflict of interest.

## Supporting information



Supporting Information

## Data Availability

The data that support the findings of this study are available in the supplementary material of this article.
