# Supplementary material for: Li+ Diffusion in LinCoNb2O6 (0 < n ≤ 6) Anode with High Capacity Density: Fast Kinetics and Mechanistic Insights
Source: Adv Sci (Weinh). 2025 Mar 19;12(18):2416001. doi: 10.1002/advs.202416001 (PMC12079548; doi:10.1002/advs.202416001)
Supplement: Supplementary file 1 — Supporting Information [file ADVS-12-2416001-s001.docx]

**Supporting Information**

**1 Experimental section**

1.1 Material preparation

CoCl_2_·6H_2_O and excess CO(NH_2_)_2_ were added to a beaker containing 30 mL of ethanol. The mixture was stirred via a magnetic stirrer until a clear solution formed. Separately, NbCl_5_ was dissolved in 10 mL of ethanol and then added dropwise to the beaker. The resulting mixture was transferred into a Teflon-lined autoclave for hydrothermal reactions at 160 °C, 180 °C, and 200 °C for 24 h, followed by cooling to room temperature. The hydrothermal products were washed 5 times with ethanol and subsequently dried in an oven. The dried samples were transferred to a crucible and heated in a muffle furnace at a ramp rate of 2 °C min^−1^ to 800 °C, maintaining this temperature for 6 h. The final CoNb_2_O_6_ products were labeled CoNb_2_O_6_-MR, CoNb_2_O_6_-MC, and CoNb_2_O_6_-MP.

1.2 Physical characterization

The phase characterization of CoNb_2_O_6_ was performed via a Rigaku MiniFlex X-ray diffractometer (XRD) with Cu-Kα radiation (λ=0.1542 nm). The morphology of CoNb_2_O_6_ was analyzed by scanning electron microscopy (SEM) with a Hitachi SU8010 instrument. The composition of CoNb_2_O_6_ was examined via X-ray photoelectron spectroscopy (XPS, Thermo Scientific Escalab 250Xi).

1.3 Battery assembly

The working electrodes were prepared by mixing the active material (CoNb_2_O_6_-MR, CoNb_2_O_6_-MC, or CoNb_2_O_6_-MP, 70 wt%), acetylene black (20 wt%), and polyvinylidene fluoride (PVDF, 10 wt%) in N-methyl-2-pyrrolidone. The mixture was stirred thoroughly to form a slurry, which was then coated onto copper foil and dried in an oven at 65 °C for 6 h. The active material loading for the prepared working electrodes with a diameter of 12 mm and a thickness of 25 μm was ~1.2 mg cm^−2^.

The coin cells for testing were assembled in an argon-filled glove box (H_2_O, O_2_ < 0.5 ppm) with lithium metal foil (a thick of 0.45 mm and a diameter of 15.6 mm, 99.99 wt%) as the counter electrode, a Celgard 2400 polypropylene membrane as the separator, and 40 µL of electrolyte consisting of 1 M LiPF_6_ in ethylene carbonate/dimethyl carbonate (1:1, v/v) with a 5 wt% fluoroethylene carbonate additive.

1.4 Electrochemical measurements

The temperature for all the electrochemical tests was maintained at 25 °C.

The galvanostatic charge/discharge tests of the assembled coin cells were conducted via a LAND battery testing system with the current densities ranging from 0.1 mA cm^−2^ to 5 mA cm^−2^ and a voltage window of 3.0−0.02 V. The capacity density and current density were calculated based on the mass of the active material, and the apparent area of the working electrode, respectively, and 1 C = 0.467 mA cm^−2^ = 472 mA g^−1^.

Cyclic voltammetry (CV) tests were performed on a VMP3 electrochemical workstation at scan rates ranging from 0.1−1 mV s^−1^, with a voltage range of 3.0−0.02 V. The current and scan rate response was obtained via the power law formula (Equation S1), and the pseudocapacitive behavior was calculated via Equation S2. The Li^+^ diffusion coefficient (*D*_Li_) was determined according to Equation S3.

| $i=av^{b}$ | (S1) |
| --- | --- |
| $i=k_{1}v+k_{2}v^{0.5}$ | (S2) |
| $D_{\mathrm{Li}}=\frac{4}{\pi\tau}\left( \frac{n_{m}V_{m}}{S} \right)^{2}\left( \frac{\Delta E_{s}}{\Delta E_{t}} \right)^{2}$ | (S3) |

Electrochemical impedance spectroscopy (EIS) measurements were also carried out via a VMP3 electrochemical workstation, with an applied AC voltage frequency ranging from 10^5^ to 10^−2^ Hz and an amplitude of 5 mV.

The galvanostatic intermittent titration technique (GITT) tests were conducted using the LAND battery testing system. For the low current density, the conditions were 0.1 mA cm^−2^ with a 5 min pulse time and a 10 min relaxation time. For the high current density, the conditions were 0.5 mA cm^−2^ with a 2 min pulse time and a 30 min relaxation time.

1.5 Theoretical calculations

The bond valence (BV) theory, which is based on Pauling’s electrovalence rule, was developed into a crystal structure theory, which has been validated by extensive experimental data. Later, I. David Brown, S. Adams, and others further advanced this theory, developing the Bond Valence Site Energy (BVSE) algorithm and software for calculating ion transport pathways and diffusion barriers based on bond valence calculations [1]. In this section, the Li^+^ diffusion pathways within the crystal structure were calculated via the BVSE method implemented via the SoftBV program [2]. BVSE is a semiempirical algorithm used to compute lithium ion site energies in material systems, enabling rapid calculation of lithium diffusion channels [3]. Visualization techniques were used to represent the possible positions of lithium ions and their potential migration pathways with yellow isosurfaces, providing an intuitive understanding of ion dynamic behavior [4]. For the calculations, a cutoff radius of 10 Å and a grid resolution of 0.1 Å were applied.

Density functional theory (DFT) calculations were performed via the Quantum Espresso (QE) software package [5]. The calculations utilized the Perdew-Burke-Ernzerhof (PBE) exchange-correlation functional, along with Grimme's DFT-D3 dispersion correction [6]. A Hubbard U correction of 3.6 eV was applied to Co, and 8.3 eV was applied to Nb. The kinetic energy cutoffs for the wavefunction and augmented charge density were set at 40 Ry and 400 Ry, respectively. Structural relaxation was performed via the Broyden-Fletcher-Goldfarb-Shanno (BFGS) method, with a force convergence threshold of 10^−3^ Ry/Bohr and an energy convergence threshold of 10^−5^ Ry. Methfessel-Paxton smearing was used with a smearing width of 0.05 Ry.

The migration energy barriers for Li^+^ diffusion were calculated via the nudged elastic band (NEB) method [7]. All the crystal structure visualizations were generated via VESTA software [8]. The formation energy of Li_x_CoNb_2_O_6_ was calculated from the total energy values via Equation S4 [9].

| $f=E\left( \text{L}\text{i}_{x}{C\mathrm{oNb}}_{2}O_{6} \right)-\frac{6-x}{6}E\left( \text{CoNb}_{2}O_{6} \right)-\frac{x}{6}E\left( \text{L}\text{i}_{6}{C\mathrm{oNb}}_{2}O_{6} \right)$ | (S4) |
| --- | --- |

The average voltage was calculated with Equation S5 [10].

| $V=-\frac{E\left( \text{L}\text{i}_{x_{1}}{C\mathrm{oNb}}_{2}O_{6} \right)-E\left( \text{L}\text{i}_{x_{2}}{C\mathrm{oNb}}_{2}O_{6} \right)-(x_{1}-x_{2})E\left( \text{Li} \right)}{\left( x_{1}-x_{2} \right)e}$ | (S5) |
| --- | --- |

1.6 Statistical analysis

In this work, the sample size for the vast majority of the data was n=1. Except for the samples with n=1, the sample size for each experiment would be clearly indicated in the figure legends. Specifically, for the CV experiments at different scan rates in Fig. 1e, the sample size was n=5. The b values were obtained from the current at the oxidation and reduction peaks and the scan rate, with a sample size of n=5 in Fig. 1f. According to the fitting, the standard deviation of the b-value at the reduction peak was ± 0.01175, with R² = 0.99872, and the standard deviation of the b-value at the oxidation peak was ± 0.01117, with R² = 0.99925.

The energy of different lithiation sites at various lithium concentrations was calculated for the convex hull plot of Co_4_Nb_8_O_24_, with the lowest energy corresponding to the most favorable lithiation site. The calculation included a total sample size of n=184. The sample sizes for each Li concentration as follows: 1Li, n=18; 2Li, n=8; 3Li, n=22; 4Li, n=12; 5Li, n=8; 6Li, n=10; 7Li, n=10; 8Li, n=10; 9Li, n=7; 10Li, n=8; 11Li, n=4; 12Li, n=4; 13Li, n=12; 14Li, n=8; 15Li, n=7; 16Li, n=8; 17Li, n=6; 18Li, n=5; 19Li, n=5; 20Li, n=5; 21Li, n=4; 22Li, n=1; 23Li, n=1; 24Li, n=1. Data analysis was performed using Origin software.

**2 Supplementary figures**

**
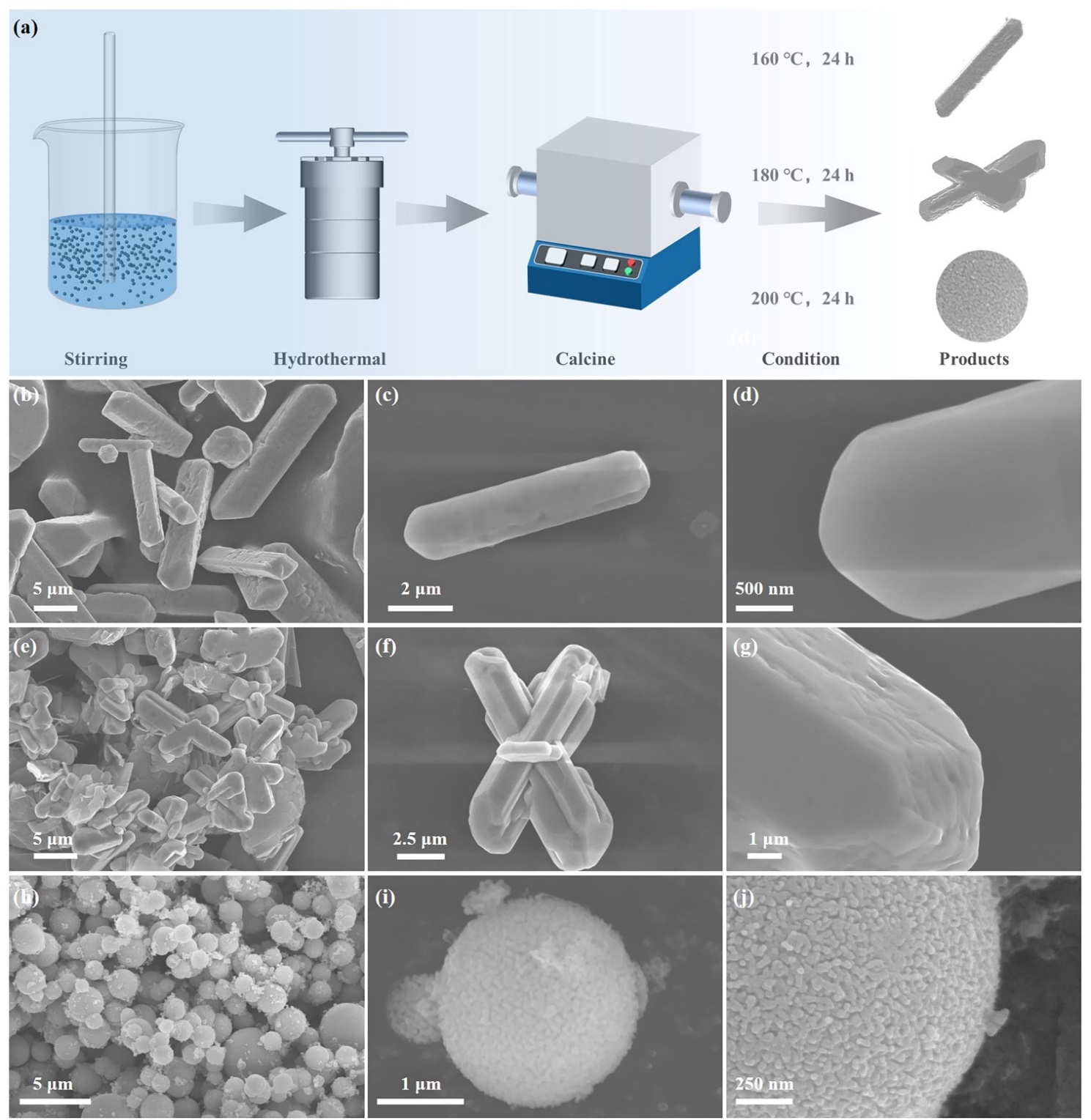
**

Figure S1 (a) Schematic diagram of CoNb_2_O_6_ synthesis and SEM images of (b−d) CoNb_2_O_6_-MR, (c−g) CoNb_2_O_6_-MC, and (h−j) CoNb_2_O_6_-MP.

**
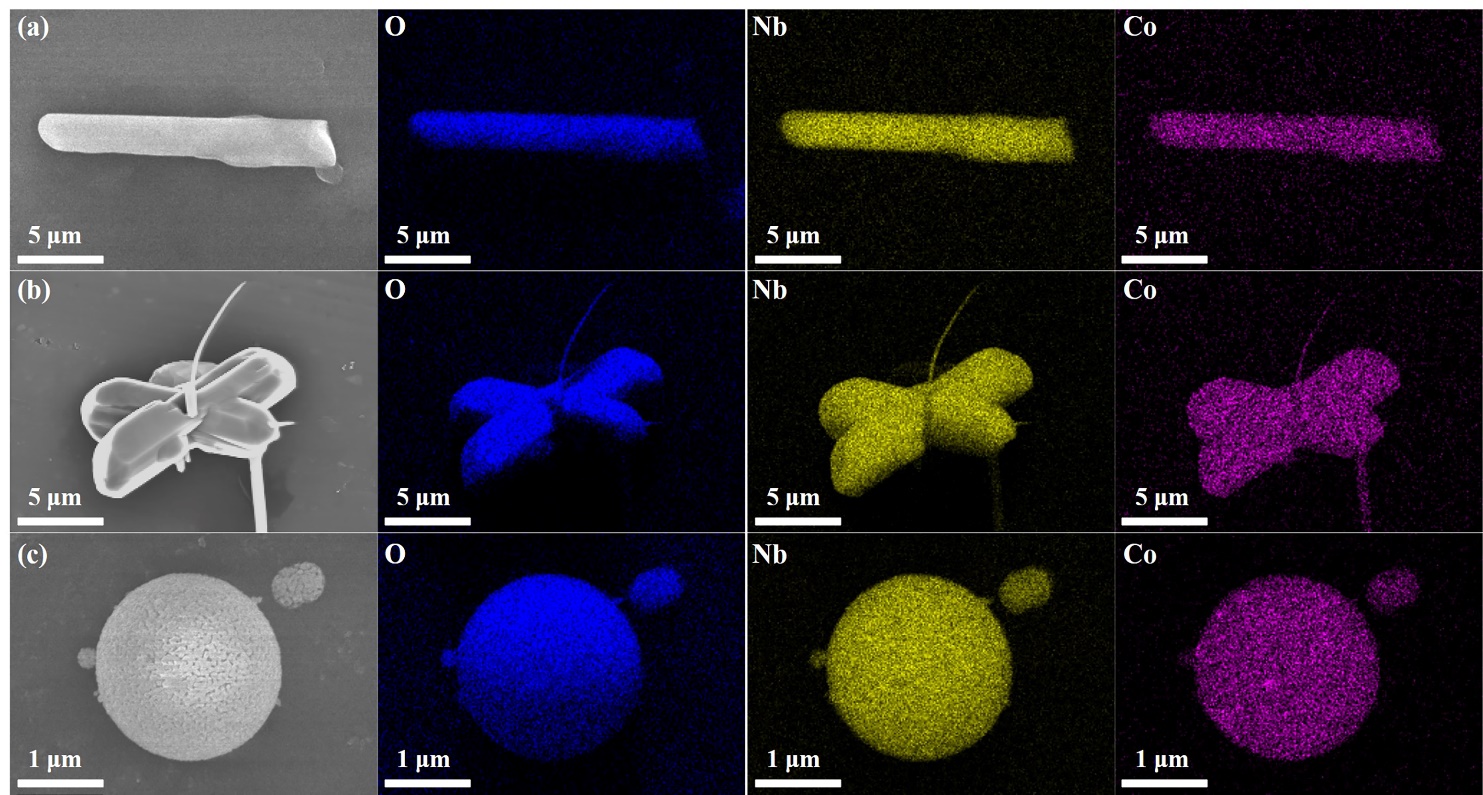
**

Figure S2 SEM images and elemental mapping of (a) CoNb_2_O_6_-MR, (b) CoNb_2_O_6_-MC, and (c) CoNb_2_O_6_-MP.


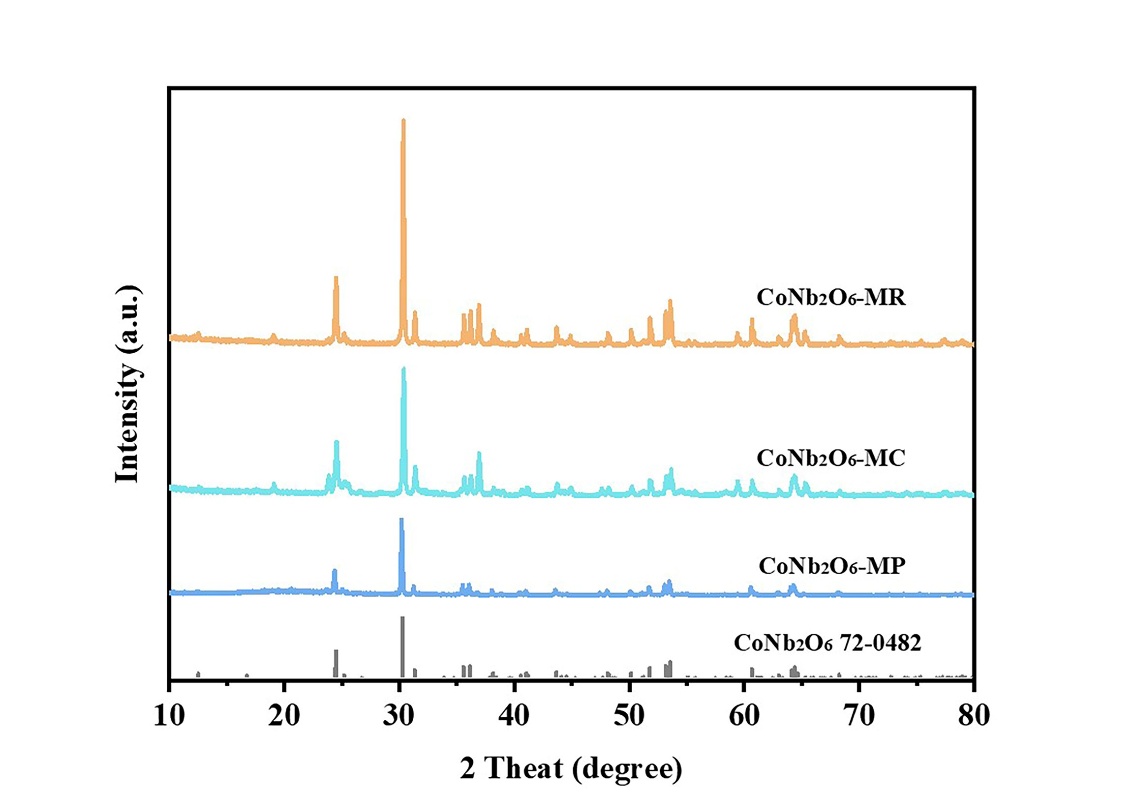


Figure S3 XRD patterns of the samples.

**
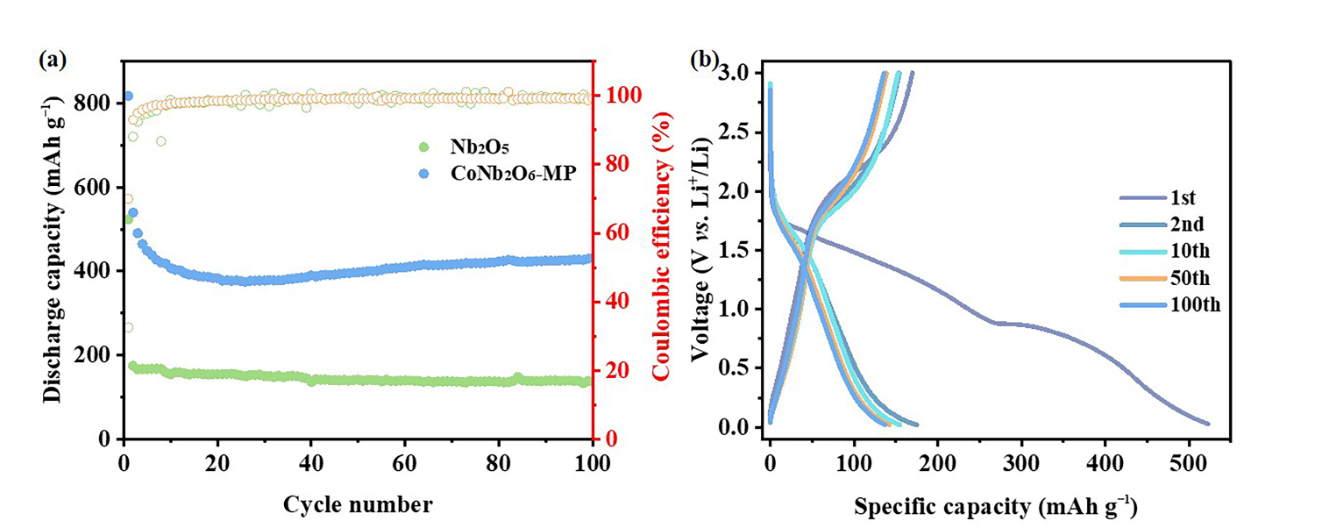
**

Figure S4 (a) Cycling performance of 0.1 mA cm^−2^ for Nb_2_O_5_ and CoNb_2_O_6_-MP and (b) charge/discharge curves of 0.1 mA cm^−2^ for Nb_2_O_5_.


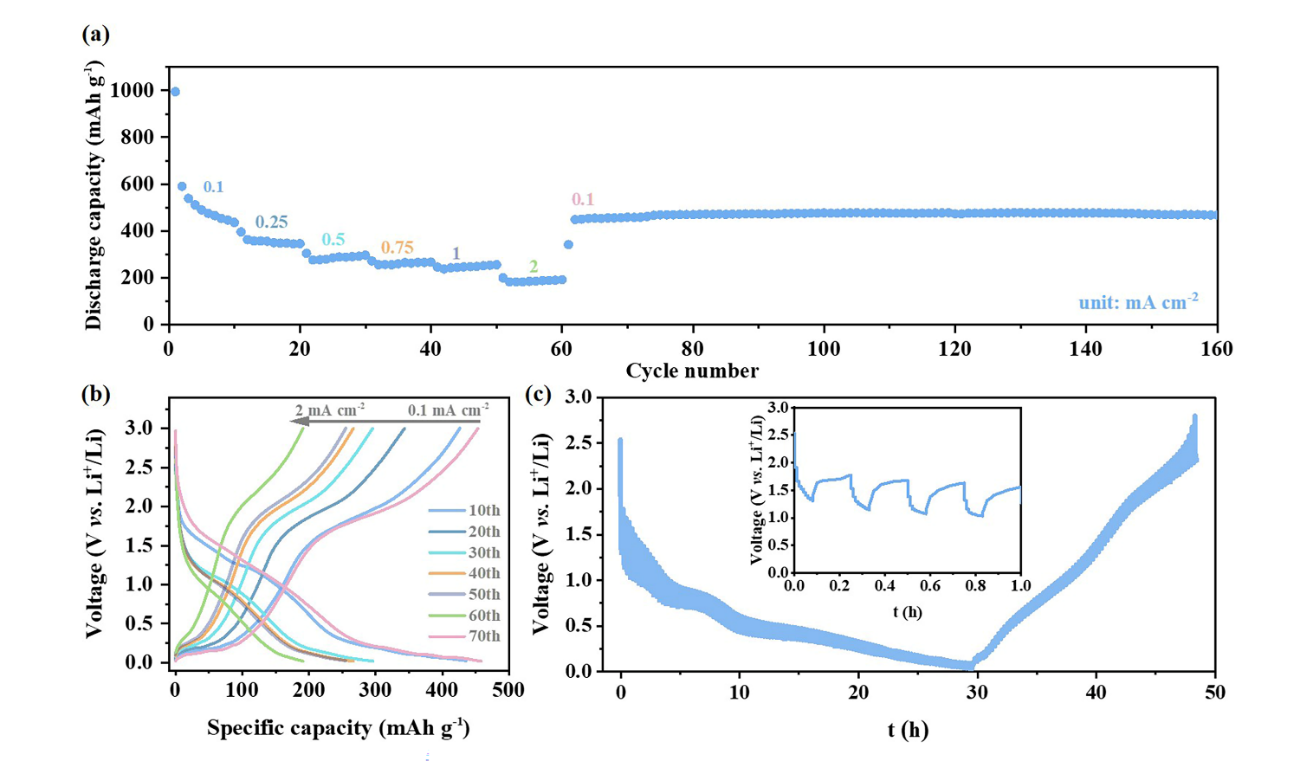


Figure S5 (a) Rate performance, (b) charge/discharge curves, (c) GITT test with 5 min pulses at a current density of 0.1 mA cm^-2^ and 10 min rest intervals of CoNb_2_O_6_-MP.

**
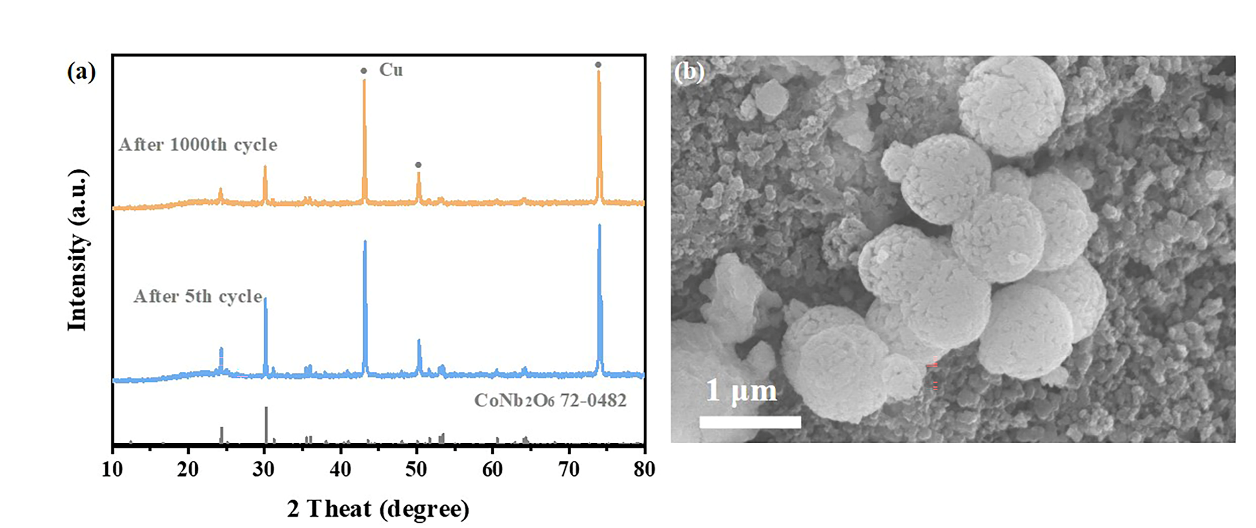
**

Figure S6 (a) XRD patterns of CoNb_2_O_6_-MP at the state of charge after the 5th cycle (0.1 mA cm^−2^) and the 1000th cycle (5 mA cm^−2^), and (b) SEM image of CoNb_2_O_6_-MP at the state of charge after the 1000th cycle (5 mA cm^−2^).

**
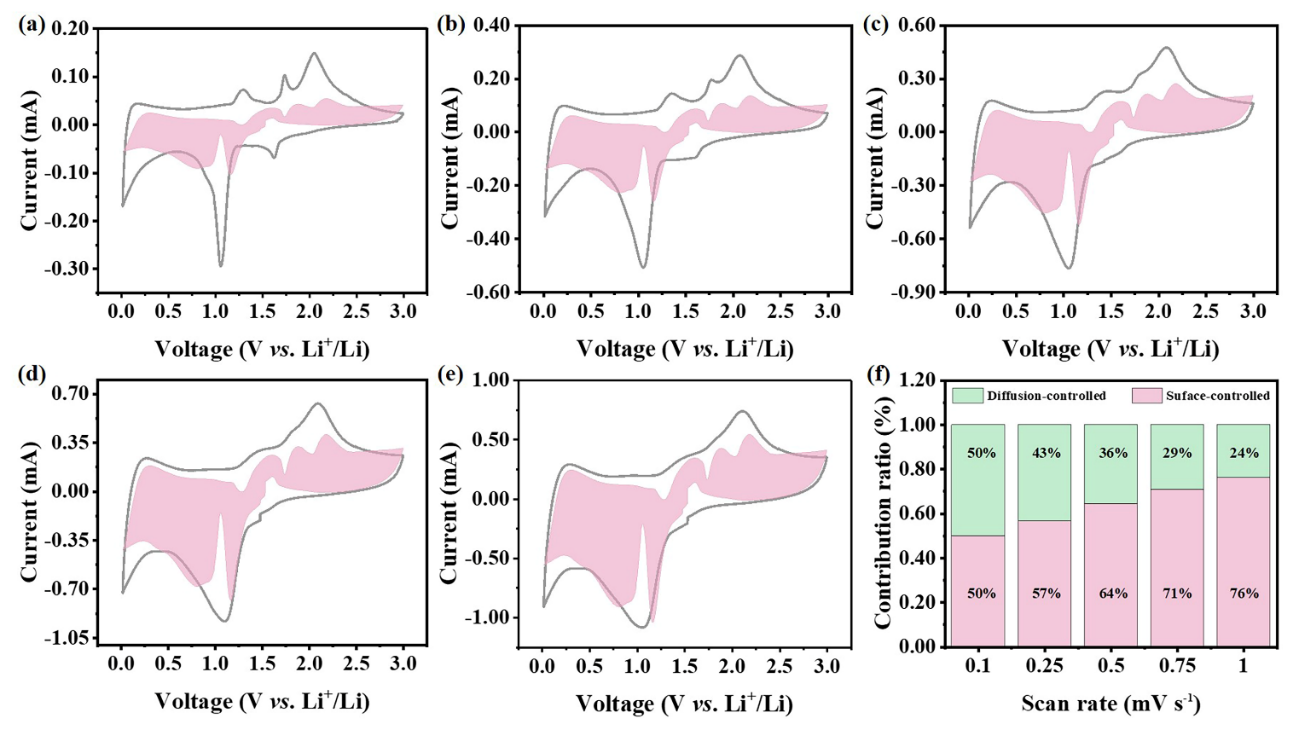
**

Figure S7 Pseudocapacitive contribution of CoNb_2_O_6_-MP with (a) 0.10, (b) 0.25, (c) 0.50, (d) 0.75, (e) 1.00 mV s^−1^ and (f) corresponding pseudocapacitive contribution ratios. As the scan rate increased, the proportion of pseudocapacitive behavior also increased, demonstrating the high efficiency and rapidity of Li^+^ intercalation/extraction in CoNb_2_O_6_-MP.

**
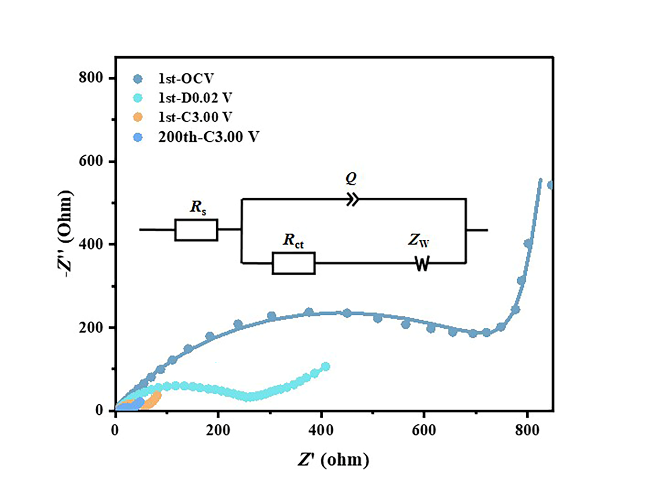
**

Figure S8 EIS tests of CoNb_2_O_6_-MP at the 1st open-circuit voltage (1st-OCV), after the first discharging with 0.1 mA cm^−2^ to 0.02 V (1st-D0.02 V), following charging with 0.1 mA cm^−2^ to 3 V (1st-C3.00 V), and in the charged state after the 200th cycle at 0.1 mA cm^−2^ (200th-C3.00 V). In the equivalent circuit diagram, *R*_s_ represents the ohmic resistance, *R*_ct_ represents the charge transfer resistance, *Z*_w_ represents the Warburg impedance, and *Q* represents the constant phase element.

**
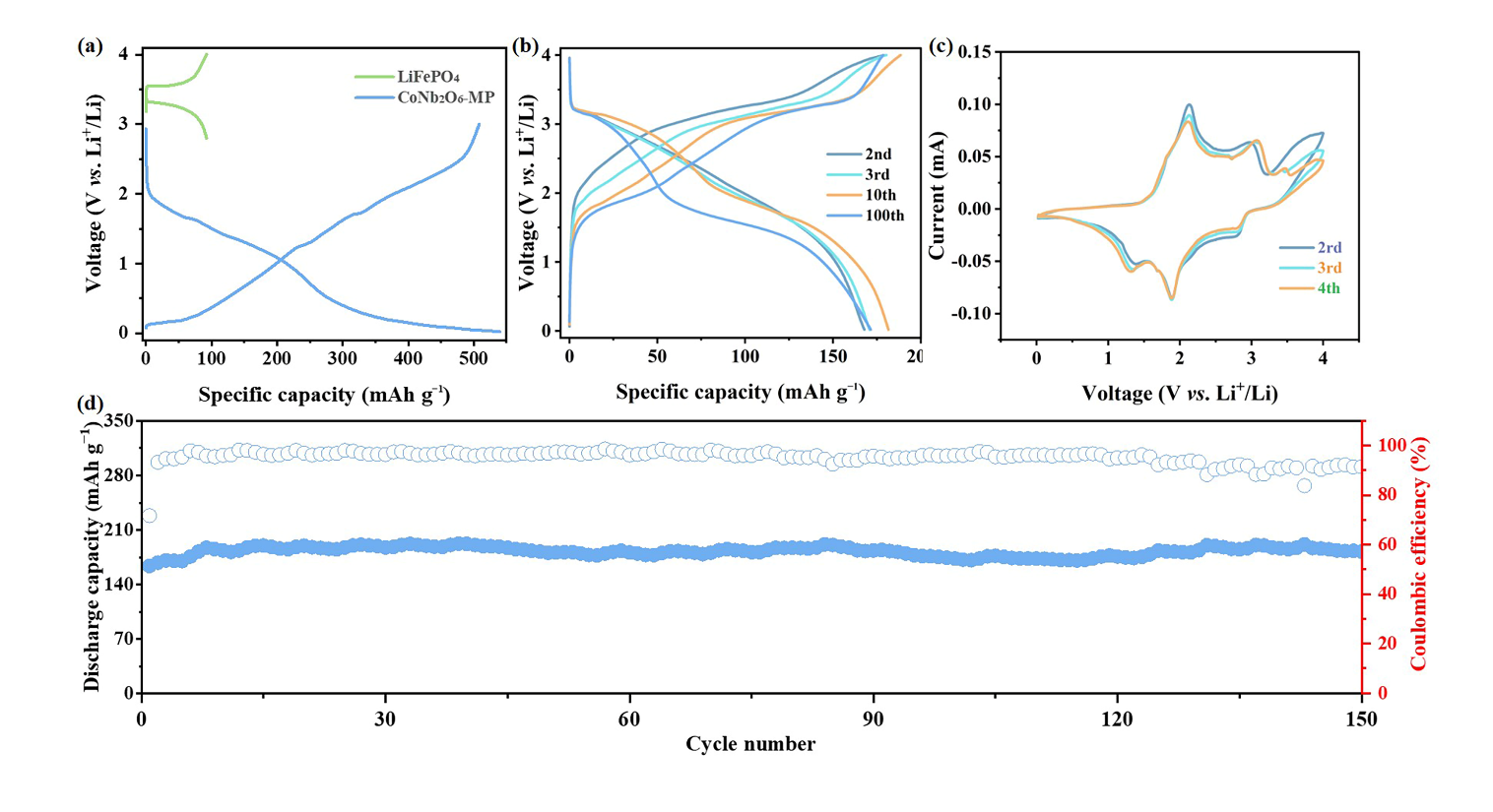
**

Figure S9 Charge/discharge curves at 0.1 mA cm^−2^ for the (a) Li||CoNb_2_O_6_-MP and Li||LiFPO_4_ batteries, (b) charge/discharge curves at 0.1 mA cm^−2^, (c) CV curves at 0.10 mV s^−1^, and (d) cycling performance at 0.1 mA cm^−2^ for the CoNb_2_O_6_-MP||LiFPO_4_ batteries.


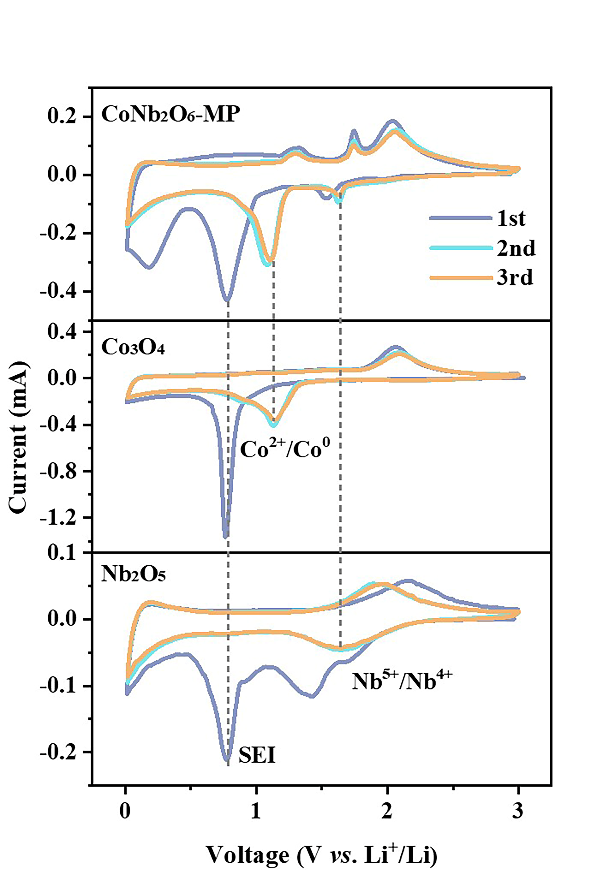


Figure S10 CV curves at 0.10 mV s^−1^ of CoNb_2_O_6_-MP, Co_3_O_4_ and Nb_2_O_5_.

**
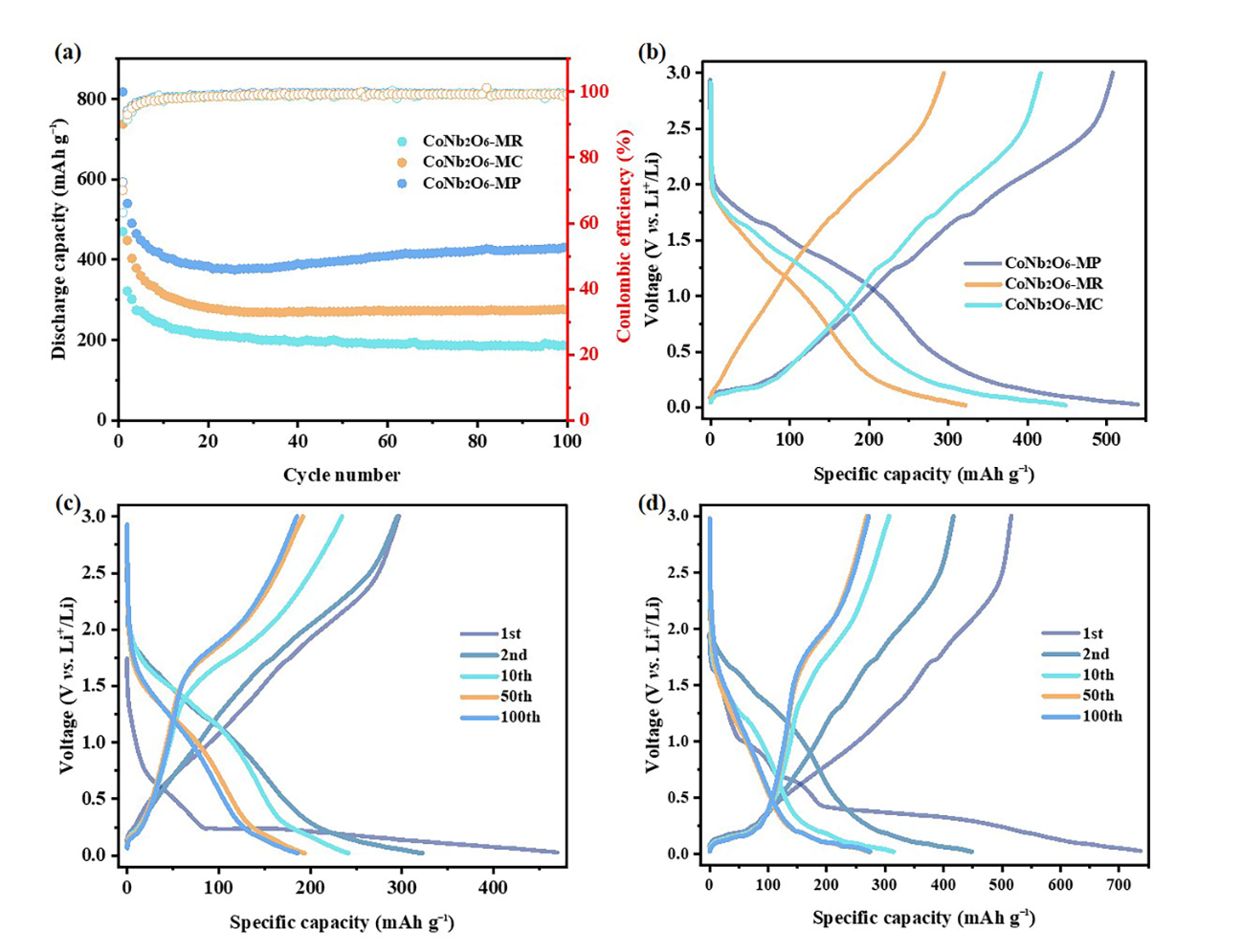
**

Figure S11 (a) Cycling performance at 0.1 mA cm^−2^ and (b) 2nd charge/discharge curves at 0.1 mA cm^−2^ for the samples and charge/discharge curves at 0.1 mA cm^−2^ for (c) CoNb_2_O_6_-MR and (d) CoNb_2_O_6_-MC.

**
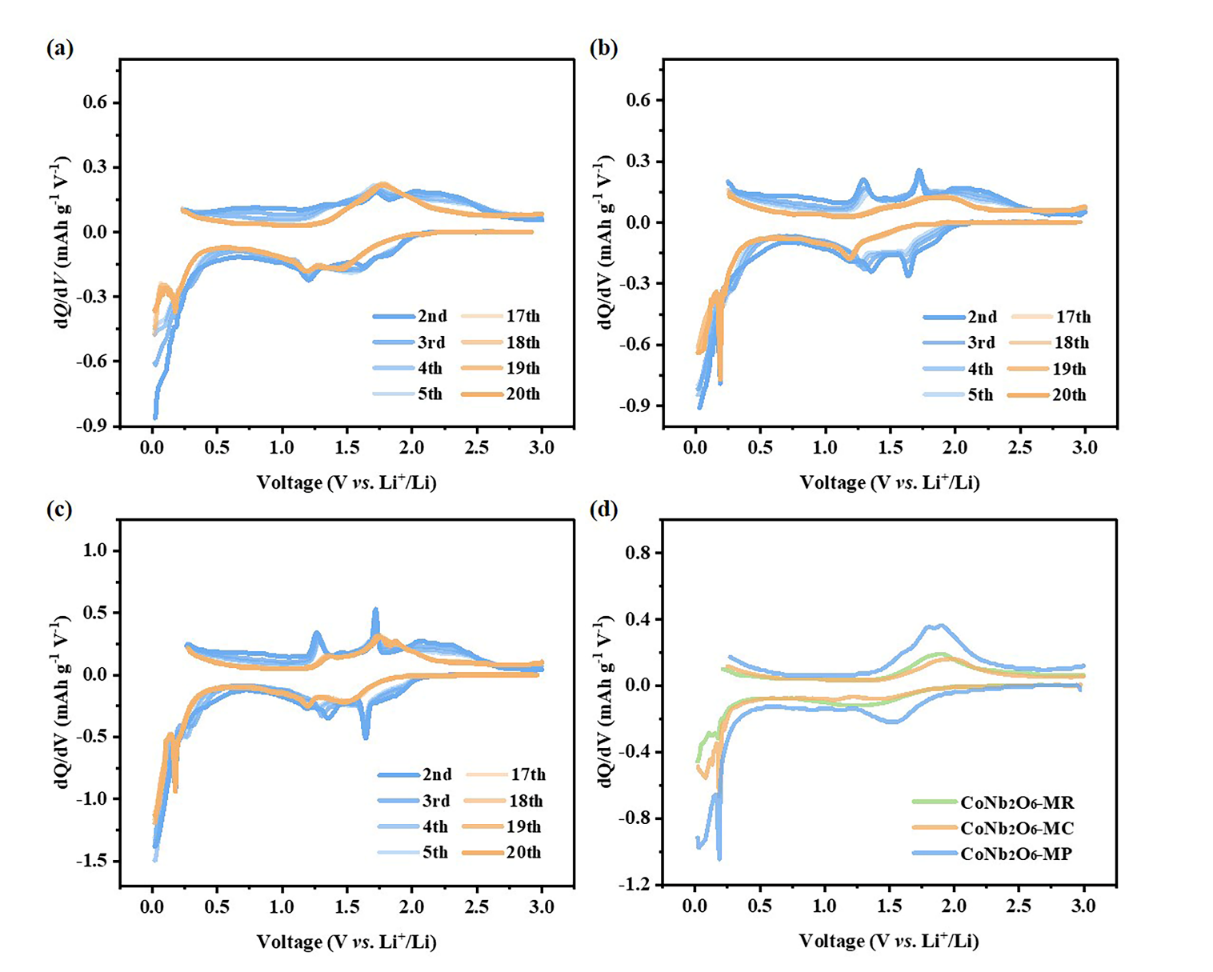
**

Figure S12 d*Q*/d*V* curves of (a) CoNb_2_O_6_-MR, (b) CoNb_2_O_6_-MC, (c) CoNb_2_O_6_-MP from charge/discharge curves at 0.1 mA cm^−2^ and (d) their 1st d*Q*/d*V* curves.


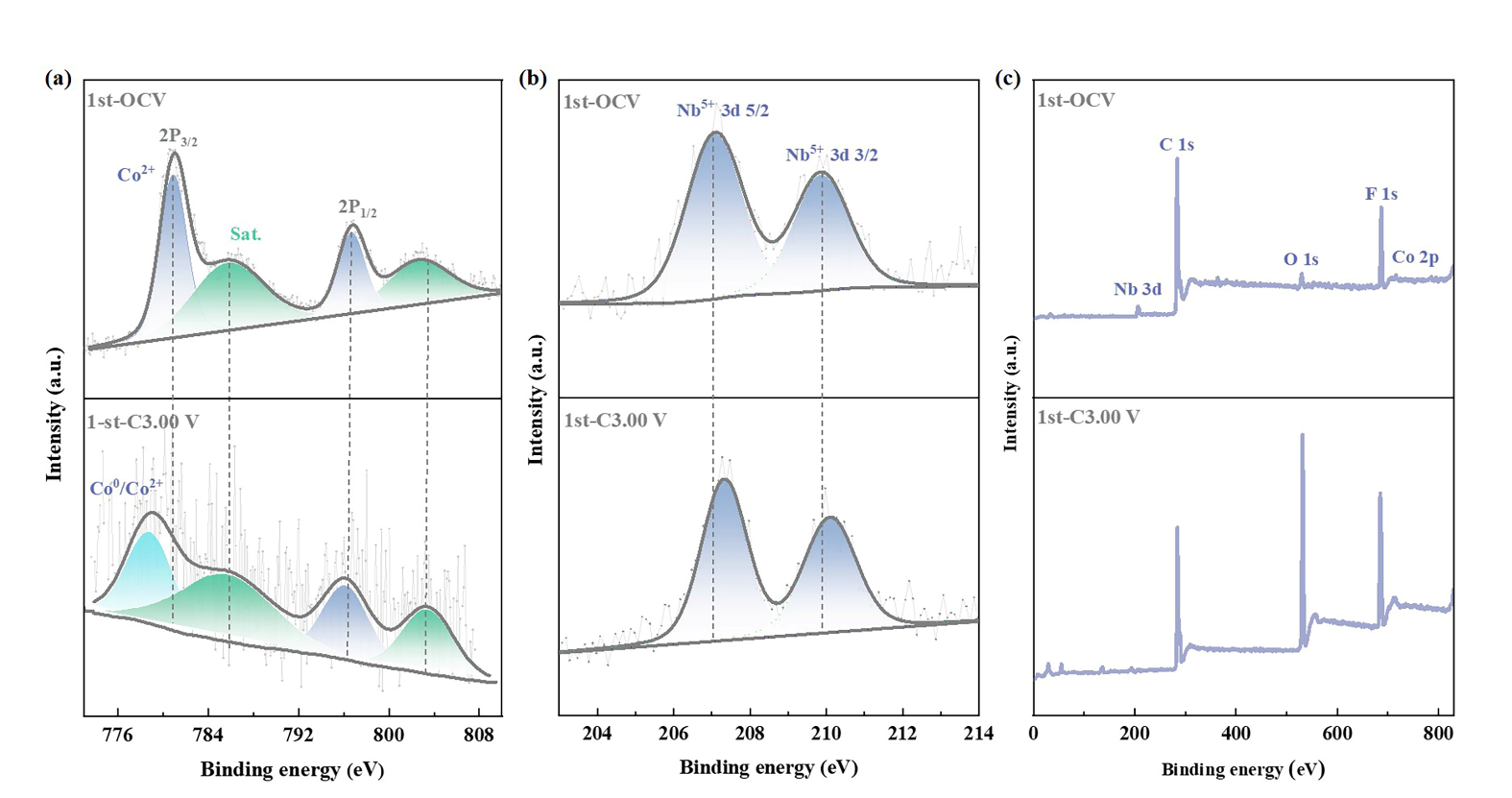


Figure S13 (a) Nb 3d spectrum, (b) Co 2p spectrum, (c) XPS survey of CoNb_2_O_6_-MP at the 1st open-circuit voltage (1st-OCV) and after charging at 0.1 mA cm^−2^ to 3 V (1st-C3.00 V).

**
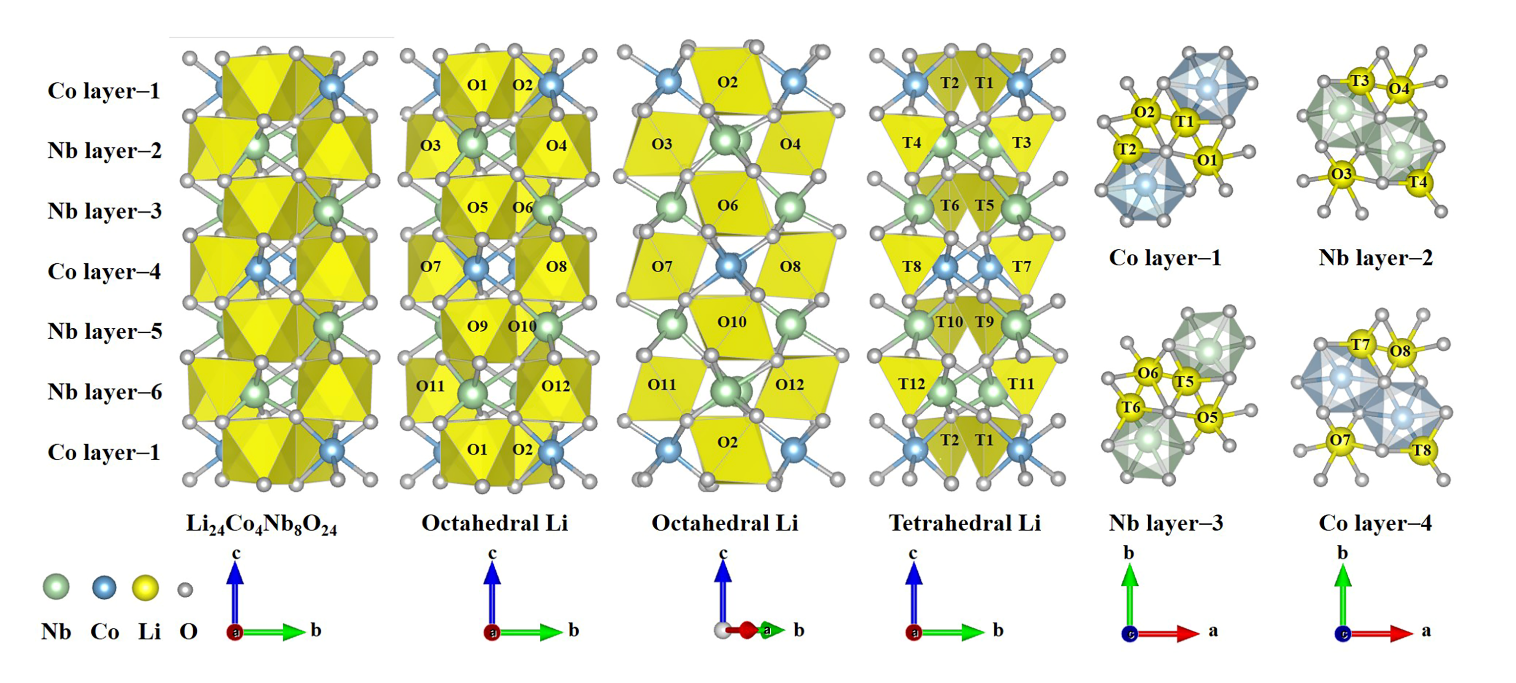
**

Figure S14 Lithium intercalation sites in the CoNb_2_O_6_ crystal.


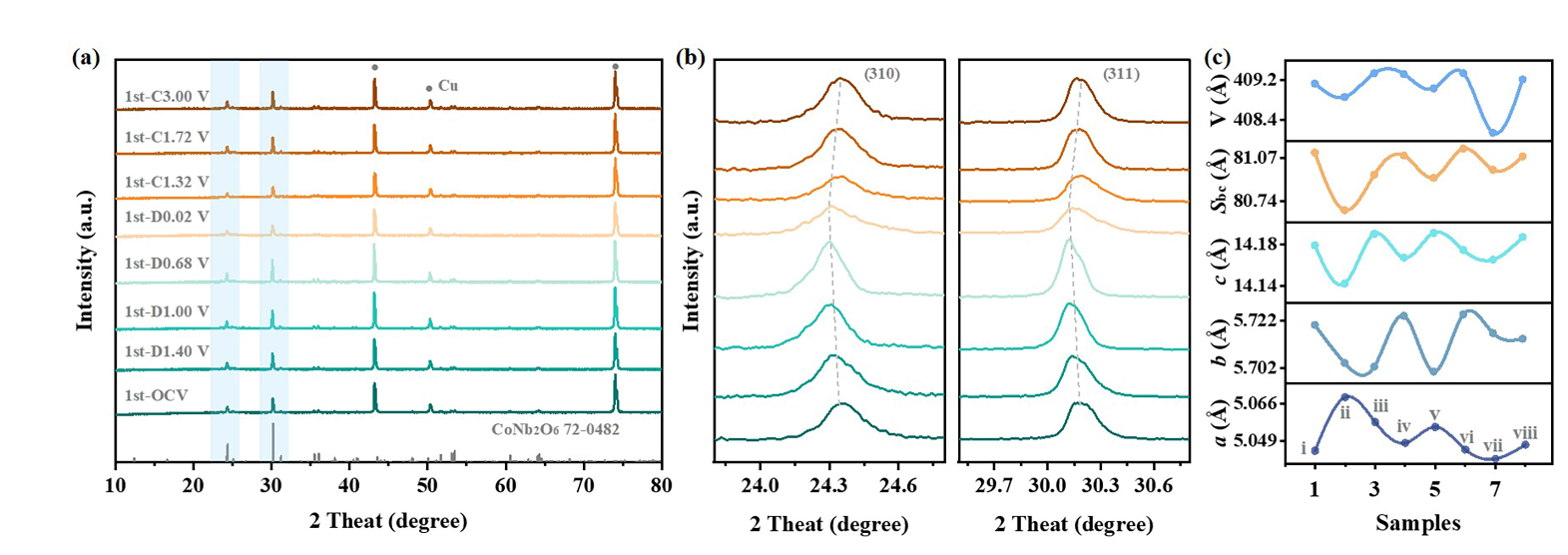


Figure S15 (a) Ex situ XRD patterns, (b) changes in the diffraction peaks of the (310) and (311) planes, (c) changes in the lattice parameters, reactive planes, and crystal volume of CoNb_2_O_6_-MP at the 1st open-circuit voltage (1st-OCV, Point i), after the first dischargeing at 0.1 mA cm^−2^ to 1.40 (1st-D1.40 V, Point ii), 1.00 (1st-D1.00 V, Point iii), 0.68 (1st-D0.68 V, Point iv), and 0.02 V (1st-D0.02 V, Point v), following charging at 0.1 mA cm^−2^ to 1.32 (1st-C1.32 V, Point vi), 1.72 (1st-C1.72 V, Point vii), and 3.00 V (1st-C3.00 V, Point viii).

**
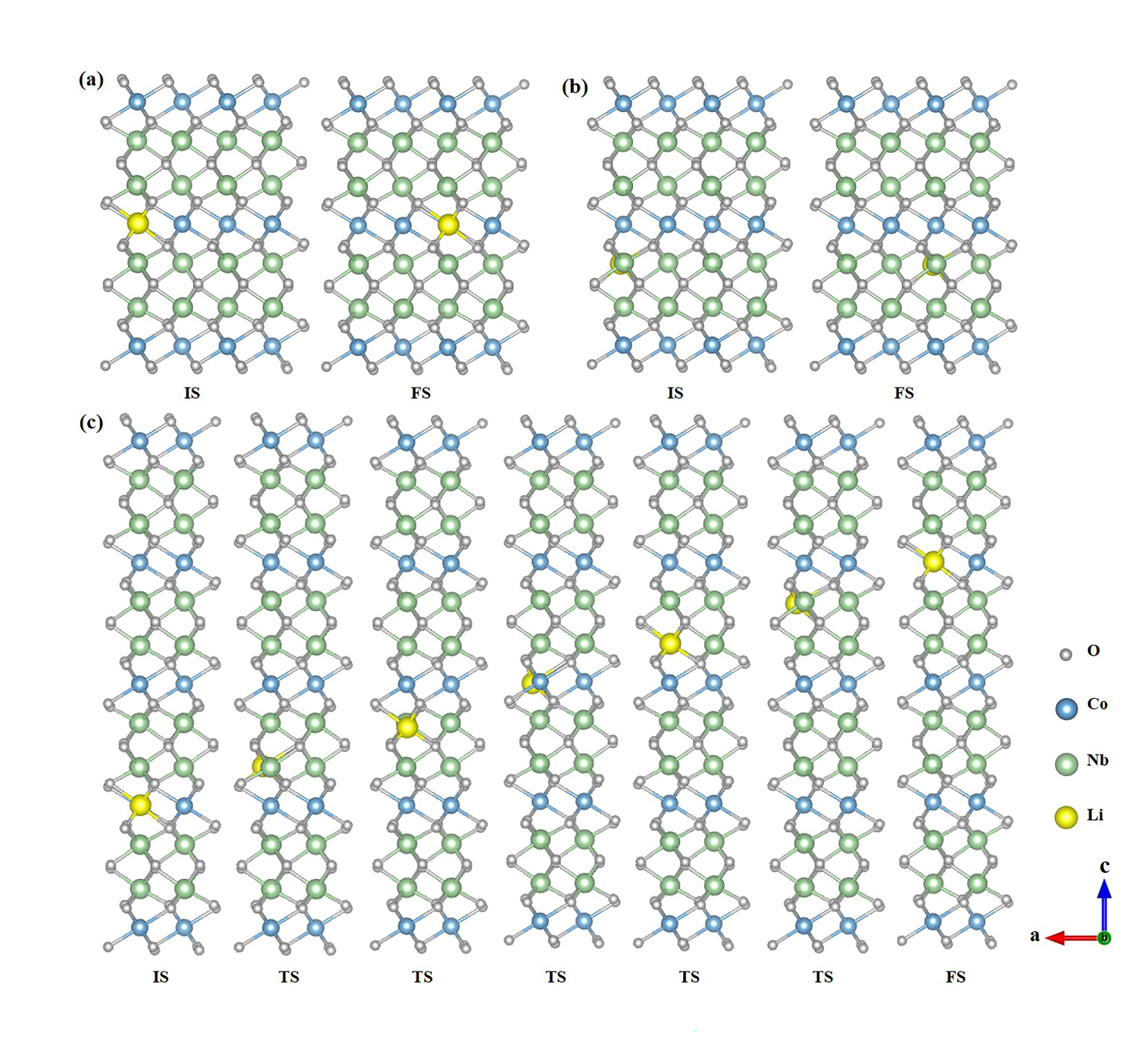
**

Figure S16 Direct-hopping diffusion mechanism at low lithium concentrations in LiCo_4_Nb_8_O_24_. (a) Initial and final states of Li^+^ diffusion along the Co layer; (b) initial and final states of Li^+^ diffusion along the Nb layer; and (c) initial, transition, and final states of Li^+^ diffusion along the c-axis.

**
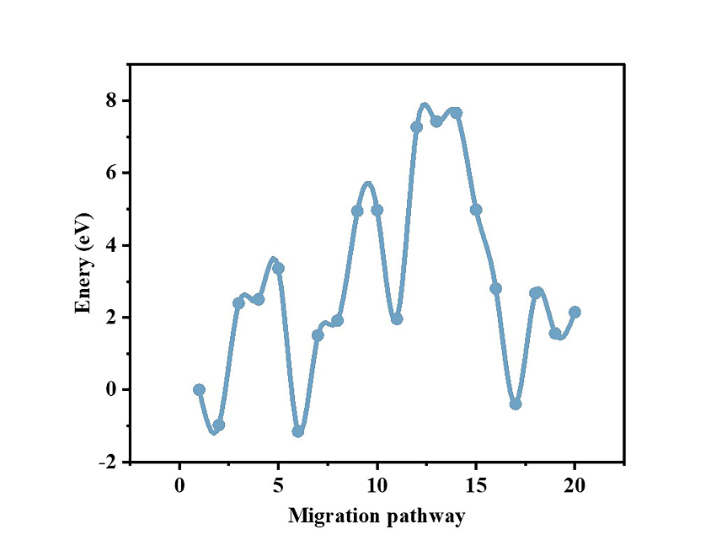
**

Figure S17 Migration barrier of Li^+^ diffusion along the c-axis.

**
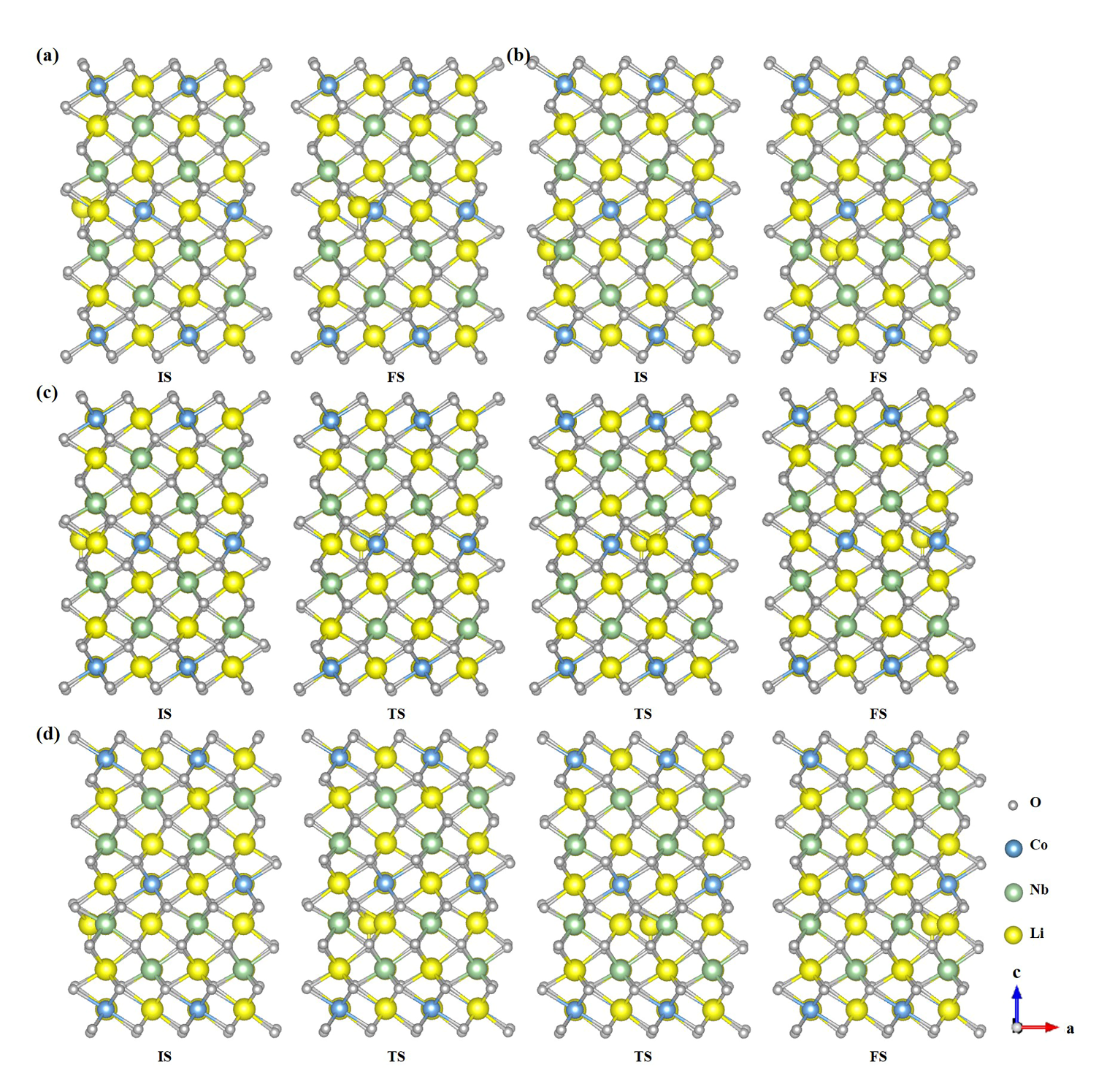
**

Figure S18 Direct-hopping and knock-off diffusion mechanisms at high lithium concentrations in Li_13_Co_4_Nb_8_O_24_. (a) Initial and final states of Li^+^ direct-hopping diffusion along the Co layer, (b) initial and final states of Li^+^ direct-hopping diffusion along the Nb layer, (c) initial, transition, and final states of Li^+^ knock-off diffusion along the Co layer, and (d) initial, transition, and final states of Li^+^ knock-off diffusion along the Nb layer.


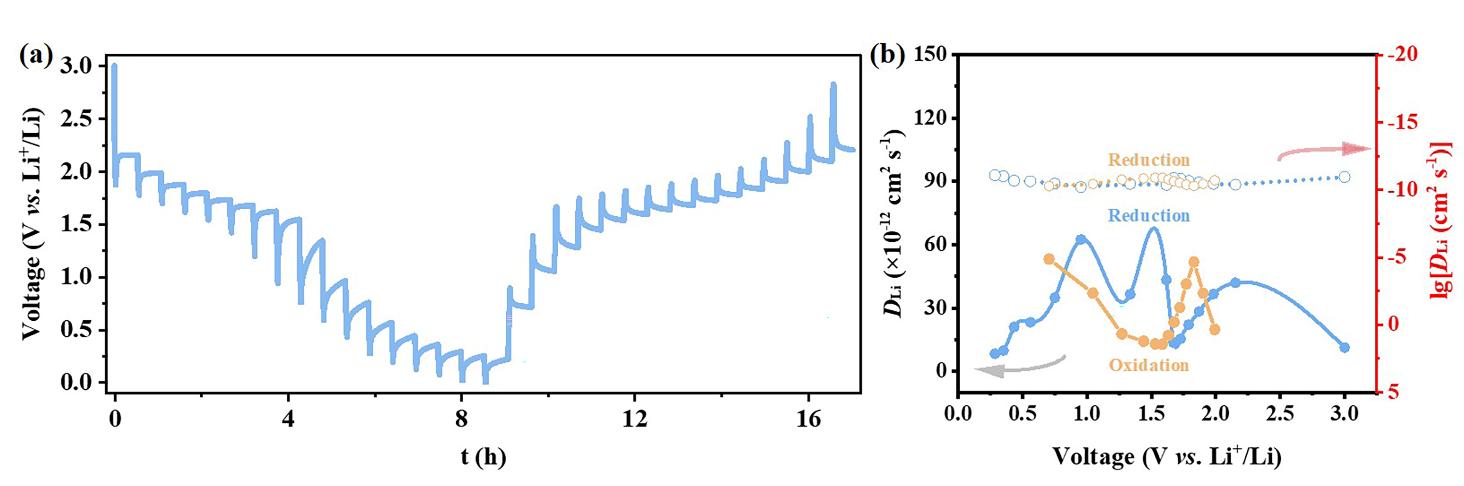


Figure S19 GITT tests of CoNb_2_O_6_-MP with 2 min pulses at a current density of 0.5 mA cm^-2^ and 30 min rest intervals.

**
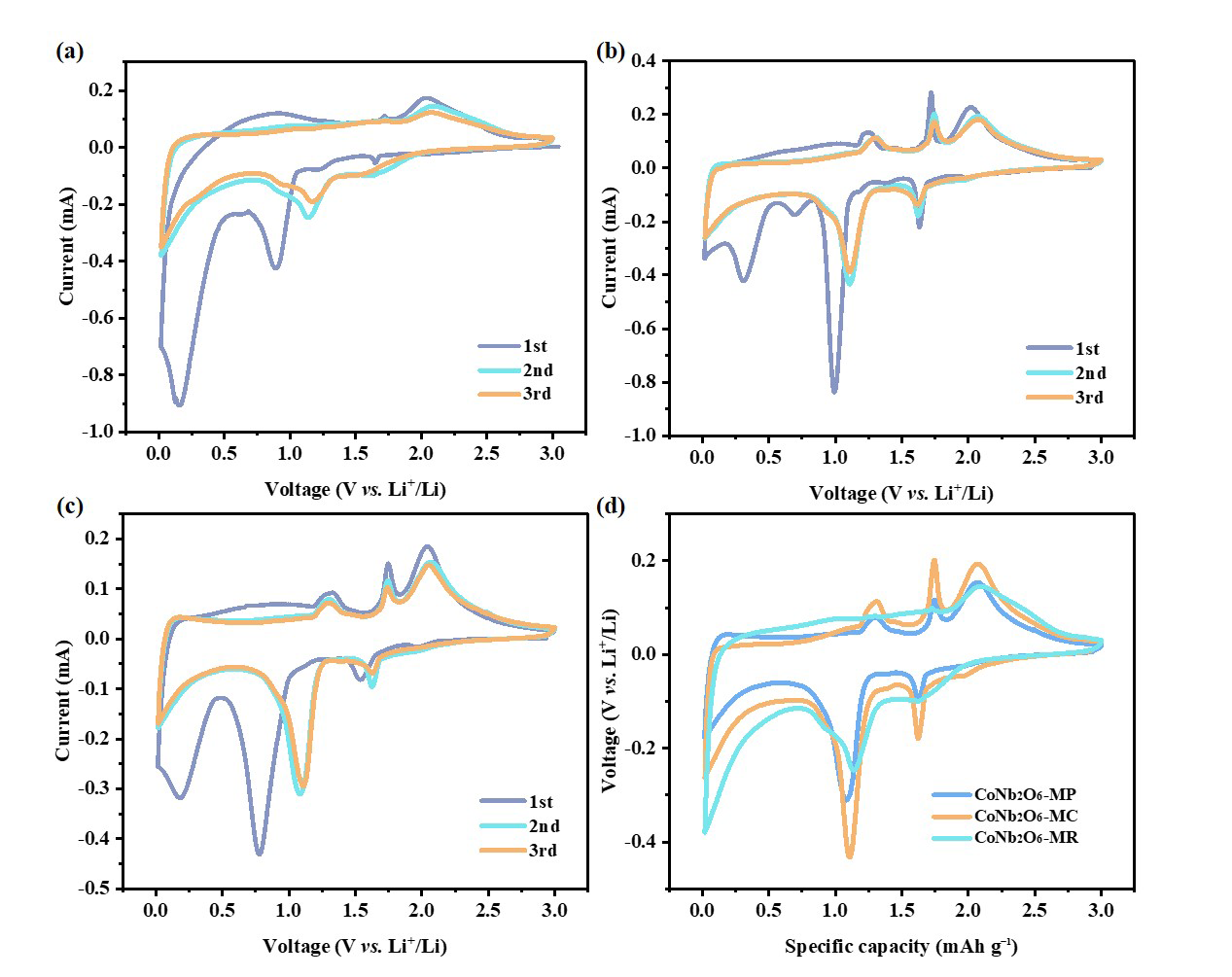
**

Figure S20 CV curves at 0.10 mV s^−1^ for (a) CoNb_2_O_6_-MR, (b) CoNb_2_O_6_-MC, and (c) CoNb_2_O_6_-MP and (d) their 1st CV curves.

**3 Supplementary tables**

Table S1 Atomic percentage of elements in CoNb_2_O_6_ obtained through EDS.

| Samples | O | Nb | Co |
| --- | --- | --- | --- |
| CoNb_2_O_6_-MR | 77.6 | 17.3 | 5.1 |
| CoNb_2_O_6_-MC | 73.2 | 19.0 | 7.8 |
| CoNb_2_O_6_-MP | 74.7 | 18.1 | 7.2 |

Table S2 EIS fitting data for CoNb_2_O_6_-MP.

| Samples | *R*_s_ | *R*_ct_ |
| --- | --- | --- |
| 1st-OCV | 3.299 | 846.80 |
| 1st-D0.02 V | 4.549 | 232.01 |
| 1st-C3.00 V | 5.516 | 58.80 |
| 200th-C3.00 V | 4.595 | 39.97 |

Table S3 Initial optimized structural model used for the theoretical calculations.

| Pbcn, *a* = 5.1324 Å, *b* = 5.6797 Å, *c* = 13.9697 Å, α=β =γ= 90° | | | |
| --- | --- | --- | --- |
| Atom type | x | y | z |
| Nb1 | 0.25485 | 0.67569 | 0.84072 |
| Nb2 | 0.75418 | 0.82748 | 0.65859 |
| Nb3 | 0.24448 | 0.67569 | 0.15882 |
| Nb4 | 0.74381 | 0.82748 | 0.34095 |
| Nb5 | 0.74381 | 0.32642 | 0.15882 |
| Nb6 | 0.24448 | 0.17463 | 0.34095 |
| Nb7 | 0.75418 | 0.32642 | 0.84072 |
| Nb8 | 0.25485 | 0.17463 | 0.65859 |
| Co1 | 0.74899 | 0.85176 | 0.00000 |
| Co2 | 0.24966 | 0.65141 | 0.49977 |
| Co3 | 0.24966 | 0.15035 | 0.00000 |
| Co4 | 0.74899 | 0.35070 | 0.49977 |
| O1 | 0.58126 | 0.60997 | 0.90698 |
| O2 | 0.08193 | 0.89320 | 0.59234 |
| O3 | 0.91672 | 0.60997 | 0.09257 |
| O4 | 0.41740 | 0.89320 | 0.40721 |
| O5 | 0.41740 | 0.39214 | 0.09257 |
| O6 | 0.91672 | 0.10891 | 0.40721 |
| O7 | 0.08193 | 0.39214 | 0.90698 |
| O8 | 0.58126 | 0.10891 | 0.59234 |
| O9 | 0.10173 | 0.89101 | 0.92034 |
| O10 | 0.60106 | 0.61215 | 0.57898 |
| O11 | 0.39760 | 0.89101 | 0.07920 |
| O12 | 0.89693 | 0.61215 | 0.42057 |
| O13 | 0.89693 | 0.11110 | 0.07920 |
| O14 | 0.39760 | 0.38996 | 0.42057 |
| O15 | 0.60106 | 0.11110 | 0.92034 |
| O16 | 0.10173 | 0.38996 | 0.57898 |
| O17 | 0.42629 | 0.87513 | 0.74259 |
| O18 | 0.92562 | 0.62804 | 0.75672 |
| O19 | 0.07304 | 0.87513 | 0.25695 |
| O20 | 0.57237 | 0.62804 | 0.24282 |
| O21 | 0.57237 | 0.12698 | 0.25695 |
| O22 | 0.07304 | 0.37407 | 0.24282 |
| O23 | 0.92562 | 0.12698 | 0.74259 |
| O24 | 0.42629 | 0.37407 | 0.75672 |

The original CoNb_2_O_6_ CIF file was obtained from the Materials Project (https://materialsproject.org) under the Creative Commons Attribution 4.0 International License.

Table S4 Lattice parameters, reactive planes, and crystal volume of CoNb_2_O_6_-MP.

| Samples | *a* (Å) | *b* (Å) | *c* (Å) | *S*_bc_ (Å^2^) | V(Å^3^) |
| --- | --- | --- | --- | --- | --- |
| 1st-OCV | 5.04425 | 5.71994 | 14.18042 | 81.11116 | 409.14515 |
| 1st-D1.40 V | 5.06903 | 5.70445 | 14.14018 | 80.66196 | 408.87783 |
| 1st-D1.00 V | 5.05753 | 5.70293 | 14.19254 | 80.93899 | 409.35107 |
| 1st-D0.68 V | 5.04790 | 5.72368 | 14.16743 | 81.0898 | 409.33305 |
| 1st-D0.02 V | 5.05526 | 5.70089 | 14.19351 | 80.91571 | 409.05013 |
| 1st-C1.32 V | 5.04478 | 5.72425 | 14.17545 | 81.1438 | 409.35236 |
| 1st-C1.72 V | 5.04052 | 5.71662 | 14.16535 | 80.97789 | 408.17052 |
| 1st-C3.00 V | 5.04708 | 5.71434 | 14.18922 | 81.08199 | 409.22755 |

Table S5 Changes in the lattice parameters, reactive planes, and crystal volume of CoNb_2_O_6_-MP.

| Samples | Δ*a* (Å) | Δ*b* (Å) | Δ*c* (Å) | Δ*S*_bc_ (Å^2^) | ΔV(Å^3^) |
| --- | --- | --- | --- | --- | --- |
| 1st-OCV | 0 | 0 | 0 | 0 | 0 |
| 1st-D1.40 V | 0.02478 | -0.01549 | -0.04024 | -0.44920 | -0.26732 |
| 1st-D1.00 V | 0.01328 | -0.01701 | 0.01212 | -0.17217 | 0.20592 |
| 1st-D0.68 V | 0.00365 | 0.00374 | -0.01299 | -0.02136 | 0.18790 |
| 1st-D0.02 V | 0.01101 | -0.01905 | 0.01309 | -0.19545 | -0.09502 |
| 1st-C1.32 V | 0.00053 | 0.00431 | -0.00497 | 0.03264 | 0.20721 |
| 1st-C1.72 V | -0.00373 | -0.00332 | -0.01507 | -0.13327 | -0.97463 |
| 1st-C3.00 V | 0.00283 | -0.0056 | 0.00880 | -0.02917 | 0.08240 |

Table S6 Comparison of the migration barriers at low lithium concentrations.

| Samples | Migration barrier (eV) | References |
| --- | --- | --- |
| Graphite | 0.420 | [11] |
| TiO_2_ | 0.496 | [12] |
| T-Nb_2_O_5_ | 0.470 | [13] |
| TT-Nb_2_O_5_ | 0.460 | [13] |
| 2D T-Nb_2_O_5_-C-rGO | 0.570 | [14] |
| WNb_2_O_8_ | 0.430 | [15] |
| W_3_Nb_14_O_44_ | 0.430 | [15] |
| W_10.3_Nb_6.7_O_47_ | 0.392 | [15] |
| Nb_14_W_3_O_44_ | 0.500 | [16] |
| NiNb_2_O_6_ | 0.460 | [17] |
| CoNb_2_O_6_ | 0.374 | This work |

**4 Supplementary references**

1. Y. Nishitani, S. Adams, K. Ichikawa, T. Tsujita, Evaluation of magnesium ion migration in inorganic oxides by the bond valence site energy method, *Solid State Ion.*, 2018, 315, 111–115. https://doi.org/10.1016/j.ssi.2017.11.031

2. H. Chen, L.L. Wong, S. Adams, SoftBV-a software tool for screening the materials genome of inorganic fast ion conductors, *Acta Cryst. B*, 2019, 75, 18–33. https://doi.org/10.1107/S2052520618015718

3. S. Adams, R.P. Rao, Understanding ionic conduction and energy storage materials with bond-valence-based methods, Springer, Berlin, Heidelberg, 2014, 158, 129–159. https://doi.org/10.1007/430_2013_137

4. K. Nie, X. Sun, J. Wang, Y. Wang, W. Qi, D. Xiao, J.-N. Zhang, R. Xiao, X. Yu, H. Li, X. Huang, L. Chen, Realizing long-term cycling stability and superior rate performance of 4.5 V-LiCoO_2_ by aluminum doped zinc oxide coating achieved by a simple wet-mixing method, *J. Power Sources*, 2020, 470, 228423. https://doi.org/10.1016/j.jpowsour.2020.228423

5. P. Giannozzi, S. Baroni, N. Bonini, M. Calandra, R. Car, C. Cavazzoni, D. Ceresoli, G.L. Chiarotti, M. Cococcioni, I. Dabo, A. Dal Corso, S. de Gironcoli, S. Fabris, G. Fratesi, R. Gebauer, U. Gerstmann, C. Gougoussis, A. Kokalj, M. Lazzeri, L. Martin-Samos, N. Marzari, F. Mauri, R. Mazzarello, S. Paolini, A. Pasquarello, L. Paulatto, C. Sbraccia, S. Scandolo, G. Sclauzero, A.P. Seitsonen, A. Smogunov, P. Umari, R.M. Wentzcovitch, Quantum espresso: a modular and open-source software project for quantum simulations of materials, *J. Phys.: Condens. Matter,* 2009, 21, 395502. https://doi.org/10.1088/0953-8984/21/39/395502

6. M. Ernzerhof, G.E. Scuseria, Assessment of the perdew-burke-ernzerhof exchange-correlation functional, *J. Chem. Phys.*, 1999, 110, 5029-5036. https://doi.org/10.1063/1.478401

7. Y. Wang, W.D. Richards, S.P. Ong, L.J. Miara, J.C. Kim, Y. Mo, G. Ceder, Design principles for solid-state lithium superionic conductors, *Nat. Mater.*, 2015, 14, 1026–1031. https://doi.org/10.1038/nmat4369

8. K. Momma, F. Izumi, Vesta: a three-dimensional visualization system for electronic and structural analysis, *J. Appl. Cryst.*, 2008, 41, 653–658. https://doi.org/10.1107/S0021889808012016

9. M.S. Islam, C.A. Fisher, Lithium and sodium battery cathode materials: computational insights into voltage, diffusion and nanostructural properties, *Chem. Soc. Rev.*, 2014, 43, 185–204. https://doi.org/10.1039/c3cs60199d

10. T. Jiang, M.L. Falk, Calculations of the thermodynamic and kinetic properties of Li_1+_*_x_*V_3_O_8_, *Phys. Rev. B*, 2012, 85, 245111. https://doi.org/10.1103/PhysRevB.85.245111

11. S. Thinius, M.M. Islam, P. Heitjans, T. Bredow, Theoretical study of Li migration in lithium-graphite intercalation compounds with dispersion-corrected DFT methods, *J. Phys. Chem. C*, 2014, 118, 2273–2280. https://doi.org/10.1021/jp408945j

12. H. Yildirim, J.P. Greeley, S.K.R.S. Sankaranarayanan, The effect of concentration on Li diffusivity and conductivity in rutile TiO_2_, *Phys. Chem. Chem. Phys.*, 2012, 14, 4565–4576. https://doi.org/10.1039/c2cp22731b

13. J. Meng, Q. He, L. Xu, X. Zhang, F. Liu, X. Wang, Q. Li, X. Xu, G. Zhang, C. Niu, Z. Xiao, Z. Liu, Z. Zhu, Y. Zhao, L. Mai, Identification of phase control of carbon‐confined Nb_2_O_5_ nanoparticles toward high‐performance lithium storage, *Adv. Energy Mater.*, 2019, 9, 1802695. https://doi.org/10.1002/aenm.201802695

14. P. Jing, K. Liu, L. Soule, J. Wang, T. Li, B. Zhao, M. Liu, Engineering the architecture and oxygen deficiency of *T*-Nb_2_O_5_-carbon-graphene composite for high-rate lithium-ion batteries, *Nano Energy*, 2021, 89, 106398. https://doi.org/10.1016/j.nanoen.2021.106398

15. W. Yao, H. Zhu, M. Wang, P. Li, P. Liu, P. Zou, A. Nie, G. Wang, F. Kang, C. Yang, Structural insights into the lithium ion storage behaviors of niobium tungsten double oxides, *Chem. Mater.*, 2022, 34, 388–398. https://doi.org/10.1021/acs.chemmater.1c03727

16. Q. Wu, Y. Kang, G. Chen, J. Chen, M. Chen, W. Li, Z. Lv, H. Yang, P. Lin, Y. Qiao, J. Zhao, Y. Yang, Ultrafast carbothermal shock synthesis of wadsley-roth phase niobium‐based oxides for fast‐charging lithium‐ion batteries, *Adv. Funct. Mater.*, 2024, 34, 2315248. https://doi.org/10.1002/adfm.202315248

17. R. Xia, K. Zhao, L.-Y. Kuo, L. Zhang, D.M. Cunha, Y. Wang, S. Huang, J. Zheng, B. Boukamp, P. Kaghazchi, C. Sun, J.E. ten Elshof, M. Huijben, Nickel niobate anodes for high rate lithium‐ion batteries, *Adv. Energy Mater.*, 2022, 12, 2102972. https://doi.org/10.1002/aenm.202102972
